# Exosomal lncRNA-H19 promotes osteogenesis and angiogenesis through mediating Angpt1/Tie2-NO signaling in CBS-heterozygous mice

**DOI:** 10.7150/thno.58410

**Published:** 2021-06-22

**Authors:** Jyotirmaya Behera, Anil Kumar, Michael J. Voor, Neetu Tyagi

**Affiliations:** 1 Bone Biology Laboratory, Department of Physiology, School of Medicine, University of Louisville, Louisville, KY 40202, USA.; 2 James Graham Brown Cancer Center, Department of Microbiology & Immunology, University of Louisville, KY 40202, USA.; 3 Departments of Orthopaedic Surgery and Bioengineering, School of Medicine and Speed School of Engineering, University of Louisville, Louisville, KY 40202, USA.

**Keywords:** lncRNA-H19 regulation, miRNA sponge, Bone formation, Angiogenesis, Extracellular vesicles

## Abstract

**Rationale:** Emerging evidence indicates that the growth of blood vessels and osteogenesis is tightly coordinated during bone development. However, the molecular regulators of intercellular communication in the bone microenvironment are not well studied. Therefore, we aim to investigate whether BMMSC-Exo promotes osteogenesis and angiogenesis via transporting lnc-H19 in the CBS- heterozygous mouse model.

**Methods:** Using RT2 lncRNA PCR array screening, we identify a bone-specific, long noncoding RNA-H19 (lncRNA-H19/lnc-H19) in exosomes derived from bone marrow mesenchymal stem cells (BMMSC-Exo) during osteogenesis. Using bioinformatics analysis, we further discovered the seed sequence of miR-106a that could bind to lnc-H19. A luciferase reporter assay was performed to demonstrate the direct binding of miR-106a to the target gene angiopoietin 1 (Angpt1). We employed an immunocompromised Nude mouse model, to evaluate the effects of BMMSC-Exo on angiogenesis *in vivo*. Using a micro-CT scan, we monitored microstructural changes of bone in the experimental mice.

**Results:** BMMSC-Exo possessed exosomal characteristics including exosome size, and typical markers including CD63, CD9, and TSD101. *In vitro*, BMMSC-Exo significantly promoted endothelial angiogenesis and osteogenesis. Mechanistic studies have shown that exosomal lnc-H19 acts as “sponges” to absorb miR-106 and regulate the expression of angiogenic factor, Angpt1 that activates lnc-H19/Tie2-NO signaling in mesenchymal and endothelial cells. Both of these effects on osteogenesis and angiogenesis are inhibited by antagonizing Tie2 signaling. Treatment of BMMSC-Exo also restored the bone formation and mechanical quality *in vivo*.

**Conclusion:** These findings provide a novel insight into how the extracellular role of exosomal lnc-H19 affects osteogenesis and angiogenesis through competing endogenous RNA networks.

## Introduction

Osteoporosis is a common metabolic bone disease characterized by low bone mass and microstructural deterioration in bone tissue. This leads to bone fragility and an increased risk of fracture 1, 2. Previously, we reported that elevated plasma homocysteine a condition called hyperhomocysteinemia (HHcy) induces skeletal loss and reduction of bone mechanical strength in mice via epigenetic changes 1. Other studies have reported an association between decreased bone mineral density (BMD) in osteoporotic postmenopausal women and high homocysteine (Hcy) levels 3, 4, 5. In the HHcy rat model, bone quality is consistently diminished in the presence of circulating Hcy 6. HHcy is a condition associated with mutation in the Cystathionine β-synthase (CBS) enzyme. However, the CBS mutation or deficiency leads to HHcy phenotype and decreased bioavailability of H_2_S 6. Previously, we also reported that CBS^+/-^ mice were associated with an increased level of Hcy in BMMSCs and subsequently metabolic bone loss 7. Patients with CBS deficiency generally have osteoporosis and an increased risk of fracture 8, 9, 10. However, gaining an understanding of the etiology of BMMSCs dysfunction in the CBS^+/-^ condition, along with developing clinical strategies to preserve skeletal health in these patients, are major challenges. A variety of strategies are used in treating skeletal abnormalities, such as a combination of scaffold materials with growth factors, cells or autologous bone transplantation, etc. Unfortunately, the above strategies could not provide an effective treatment 2, 11. Therefore, novel strategies are needed to induce bone development and to treat skeletal abnormalities.

Recently, BMMSCs have received increased attention in regenerative medicine. BMMSCs can self-renewal, differentiate into various cell types (including osteoblasts, chondrocytes, and adipocytes), and perform migration, and paracrine signaling 12-16. A recent report suggests that BMMSCs are a promising candidate for effective fracture healing and bone development 17-19. Others have shown that BMMSCs can modulate angiogenesis by enhancing vessel development from surrounding tissues and promoting regenerative capability in rabbit models of femoral head osteonecrosis 20. However, clinical transplantation of BMMSCs has several limitations due to the low survival rate and immune rejection 21. Therefore, an alternative approach is necessary beyond cellular therapy. More recently, cell culture-derived extracellular vesicles are attracting considerable interest due to their role in intercellular communication. Recent research on extracellular vesicles (exosomes, 30-100 nm; microvesicles, 50-2000 nm) has attracted attention in the field of bone regeneration 22, 23. Exosomes (Exo) are produced by almost all cell types of the human body and contain functional proteins, microRNAs (miRNAs), mRNA and non-coding RNAs (ncRNAs) 24, 25. Notably, exosomes (Exo) do not express major histocompatibility complex (MHC) I or II, thereby overcoming all the disadvantages of cell therapy 2, 23. Findings from recent studies have indicated BMMSC exosomes induced fracture healing and skeletal muscle regeneration by promoting angiogenesis in an *in vivo* and *in vitro* model 26, 27. However, the mechanism remains elusive.

In the last decade, non-coding RNAs (ncRNAs) have emerged as novel regulators of biological processes, such as transcriptional regulation and cellular differentiation 28. Studies have shown that lncRNAs compete for endogenous RNAs (ceRNAs) to sponge miRNAs, thereby repressing translations of the mRNA targets 29. The molecular mechanisms of lncRNAs through exosome cargo in regulating angiogenesis and osteogenesis in a mouse model of skeletal loss is still not well understood. This prompted us to study how BMMSC-Exo mediate cellular communication during bone development and skeletal loss in the *CBS^+/-^* mice model.

In the present study, we hypothesized that BMMSC-Exo maintain bone mass in *CBS^+/-^* mice by mediating intercellular communication to balance osteogenesis and angiogenesis. We identified lnc-H19 is enriched in BMMSC-Exo. It also functions as a novel activator of ANGPT-Tie2 axis, by serving as a miRNA-106a sponge, which eventually promoted endothelial angiogenesis and BMMSCs osteogenesis. We further evaluated the transplantation of exosomal cargo containing lnc-H19 is required to maintain bone homeostasis in CBS^+/-^ mice. Thus, our results advocate BMMSC-Exo as a promising therapeutic approach against metabolic osteoporosis.

## Results

### The CBS enzyme is involved in osteogenesis of BMMSCs and activates angiogenesis via a paracrine dependent manner *in vitro*

**Figure 1:**
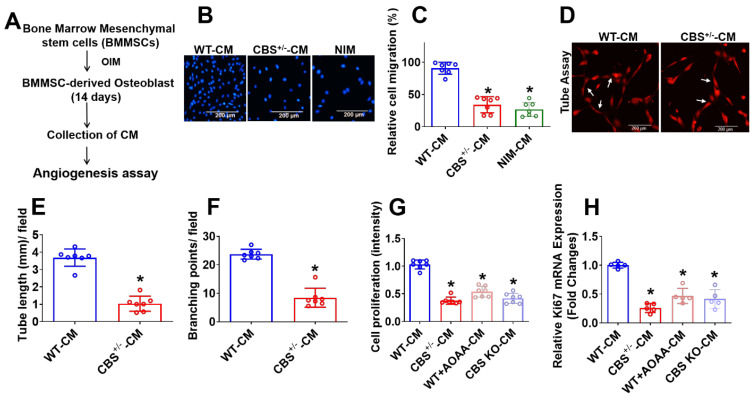
** Experimental strategies and studying the angiogenesis using conditioned media secreted during BMMSCs osteogenesis.** (A) Experimental strategy for studying conditioned medium (CM) derived from osteogenic BMMSCs and examined its effect on EC angiogenesis. BMMSCs were cultured in the osteogenesis induction medium (OIM). After 14 days, the CM was harvested and examined for effects on EC angiogenesis. (B-C) CM was applied at different experimental conditions. HUVEC migration was analyzed using a Trans-well migration assay. NIM-CM denotes non-osteogenesis induction medium-CM. Scale bar, 200 μm (D-F) HUVECs tube formation assay was performed in the presence of CM derived from experimental conditions and was stained with DiI-acLDL. Images (X40) were taken using a fluorescence microscope. The scale bar: 200µm (D). Quantification of CM-induced capillary tube formation (tube length and branching point.) (E-F). (G) *In vitro* cell, proliferation assay was measured in HUVECs culture under the presence of CM derived from various conditions. (H) qPCR analysis of mRNA transcript of Ki67 in HUVEC culture under the presence of CM derived from various conditions. Results were repeated at least three times. All data are expressed as mean±SEM. n=5-7 mice for all groups. *p < 0.05 compared with the WT-CM.

The CBS enzyme is known to regulate endothelial function 30. First, we investigated its role in BMMSCs and angiogenesis, which is crucial for bone formation. To identify undefined secretory molecules produced during osteogenesis of BMMSCs that attenuate angiogenesis, the conditioned medium (CM) were collected after 14 days of osteogenic induction in BMMSCs (Figure 1A). We then studied angiogenic properties by using a BMMSCs-endothelial cell (EC) co-culture model. Alkaline phosphatase (ALP) and alizarin red staining (ARS) was demonstrated that BMMSCs isolated from CBS^+/-^, WT+AOAA (CBS inhibitor), and CBS knockdown (KO through CRISPR/Cas9) mice. The data showed that BMMSCs isolated from these mice have less osteogenic differentiation on day 14 compared to WT control (Figure S1A-D). Further, the osteogenic gene (Runx2 and Bglap) expression was significantly reduced in BMMSCs isolated from CBS^+/-^ mice (Figure S1E) compared to BMMSCs isolated from WT mice.

After employing trans-well migration and 3D-matrigel angiogenesis assay, we next examined whether the CM from differentiated BMMSCs affected angiogenic induction. The results showed that CM from BMMSCs culture derived from CBS^+/-^ mice (CBS^+/-^-CM) as well as non-induction medium (NIM) significantly attenuated the migration of endothelial cells assessed by Trans-well assay compared to control (WT-CM) (Figure 1B-C). Matrigel was used to conduct tube formation and angiogenesis *in vitro* by detecting the number of branch points, and branch length. The results showed that the tube length and number of branch points were significantly lower in the CBS^+/-^-CM group compared to the controls (Figure 1D-F). Additionally, MTT assay showed endothelial cell proliferation attenuated in CBS^+/-^-CM (Figure 1G) compared to WT-CM. On the contrary, the CM derived from WT+AOAA-CM or CBS KO-CM resulted in a significant decrease in endothelial cell proliferation (Figure 1G) compared to controls. Further, the data also confirmed that cell migration and *in vitro* angiogenesis was significantly attenuated in WT+AOAA-CM and CBS KO-CM condition compared to WT-CM (Figure S1F, G-I). qPCR data further confirmed that the Ki67 mRNA transcript, a marker of cell proliferation, decreased in CBS^+/-^-CM compared to controls (Figure 1H). Altogether, the data reveal that loss of CBS in BMMSCs results in a significant loss of crucial endothelial phenotypes such as proliferation, migration and tube formation in the co-culture method assays (Figure 1).

### Characterization of BMMSCs and BMMSCs-derived exosomes

**Figure 2:**
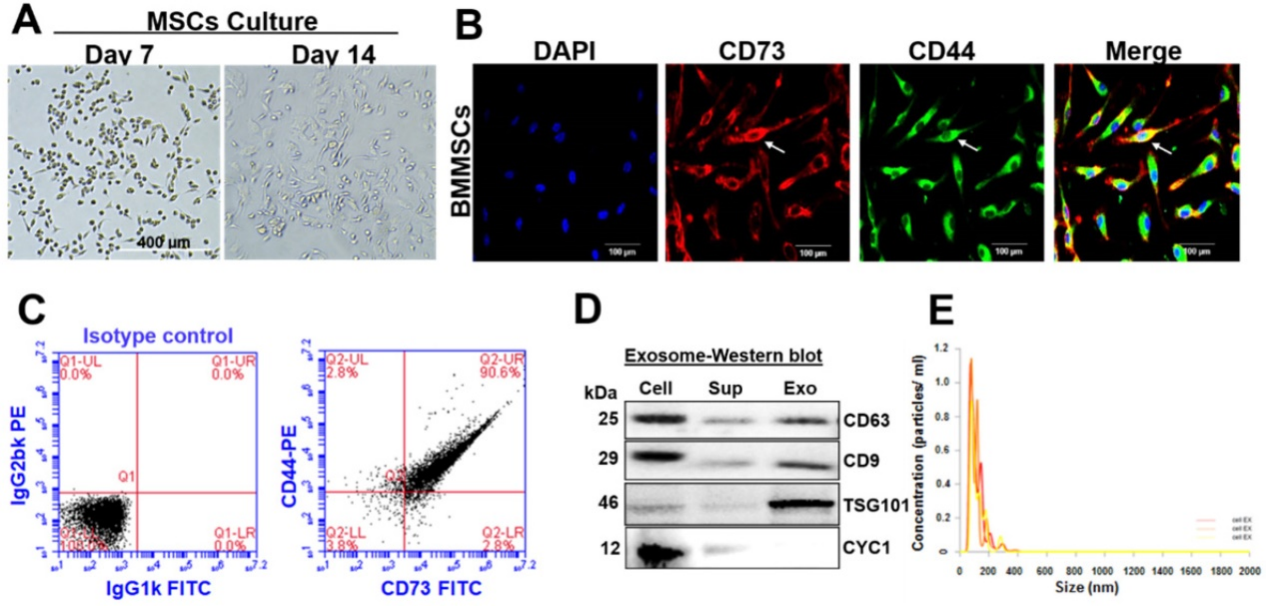
** Characterization of BMMSC and BMMSC-derived exosomes. (A)** The isolated BM was cultured under osteogenic conditions for 14 days and allowed to differentiate into BMMSCs. Scale bar, 400 µm. **(B)** Immunofluorescent staining showed that mMSCs co-expressed CD73 and CD44. Scale bar, 100 µm. **(C)** Flow cytometry analysis of BMMSCs that co-expressed CD73, and CD44 on the 14^th^ day. **(D)** Western blot analysis of protein expression of CD63, CD9, TSG101 and CYC1 from cell lysates, supernatants and exosomal contents. **(E)** Average concentration/size distribution of isolated exosomes from CM of BMMSCs, as evaluated by Zetasizer Nano ZS analysis. The results are from three independent experiments. n=5 mice for the experimental group.

Isolated bone marrow (BM) cells were cultured under an osteogenic medium for 14 days. The BMMSCs attained 20-40% confluence after seven days and 80-85% confluence after 14 days. These cells displayed a homogenous fibroblast-like morphology (Figure 2A). In this regard, immunostaining analysis confirmed that BMMSCs co-express both CD73 and CD44 (typical mesenchymal stem cell markers) (Figure 2B). FACS analysis validated the expressions of CD73 and CD44 in BMMSCs and revealed that BMMSCs co-expressed CD73 and CD44 (90.6%) (Figure 2C). Exosomes were isolated from conditioned media of BMMSCs to investigate the possible molecular mediators of BMMSCs-derived paracrine effects. Western blotting and Zetasizer Nano ZS analysis were used to determine exosomal protein expression and size distribution. Western blot analysis determined the specific presence of classical tetraspanin surface markers; CD63, and CD9 protein band in BMMSCs, as well as BMMSCs-Exo preparations (Figure 2D). Indeed, the presence of TSG101, a commonly used exosome marker, was highly enriched in the BMMSCs-Exo preparations. However, galectin-6 (Gal-6), a typical negative marker was not detected in BMMSCs-Exo (Figure 2D). The average size of exosomes mainly ranged from 80 to 120 nm, as determined by Zetasizer Nano ZS analysis (Figure 2E).

### Characterization of long non-coding RNAs (lncRNAs) profile of BMMSCs-derived exosomes

**Figure 3:**
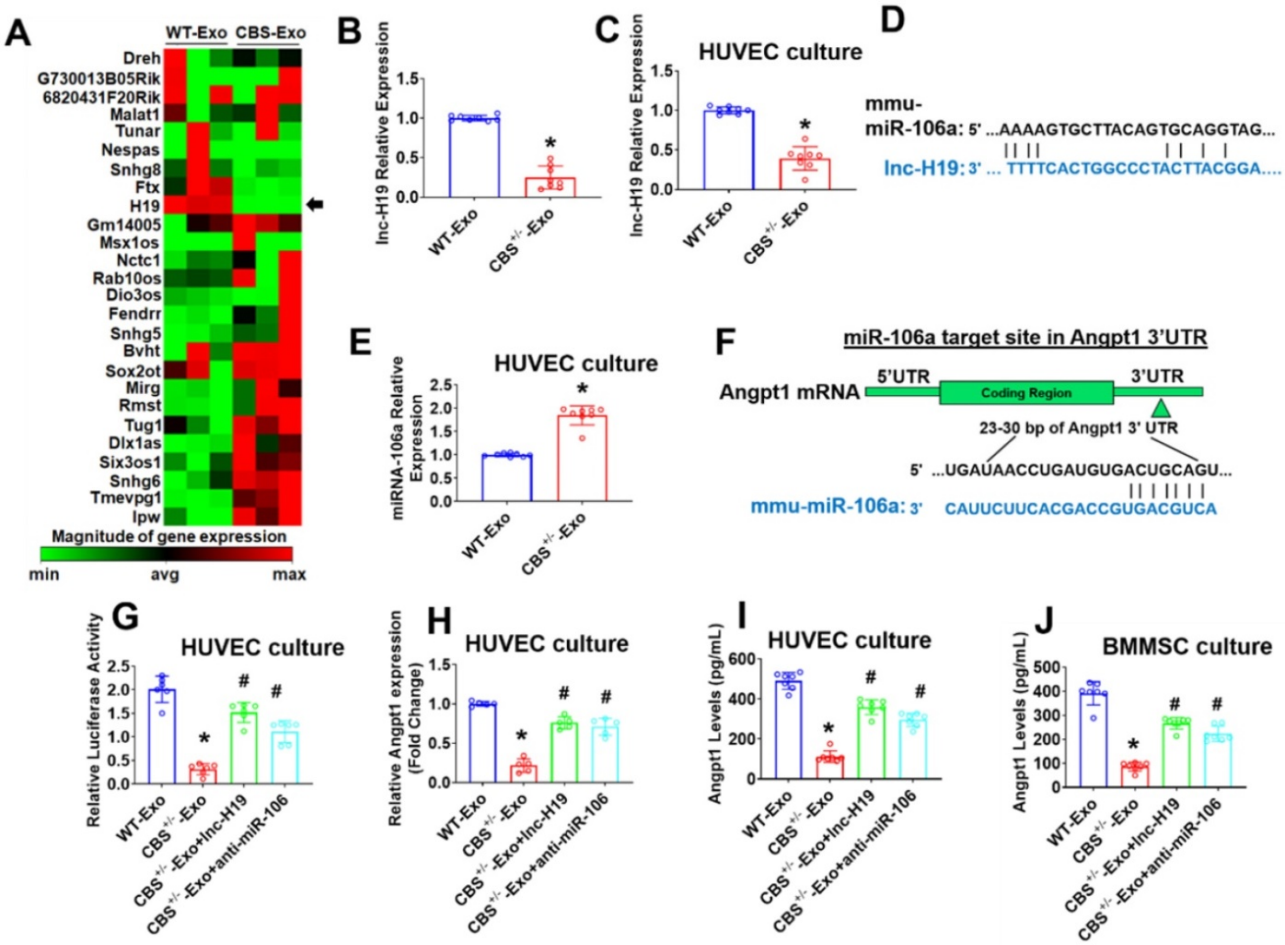
** Long non-coding RNA (lncRNA) profiling and lnc-H19 regulated miR-106a expression by functioning as a molecular sponge. (A)** Heat map of lncRNA PCR Array shows differentially expressed lncRNAs in a representative group of exosomes. The black arrow indicated the expression of lnc-H19. **(B)** The validation of lnc-H19 expression by qRT-PCR analysis. **(C)** lnc-H19 expression was performed in HUVEC culture using qPCR assay. **(D)** Schematic representation of the miR-106a binding sites in lnc-H19 using *in silico* analysis. **(E)** The expression level of miR-106a in HUVEC culture treated with CBS^+/-^-Exo by qRT-PCR analysis. **(F)** Schematic representation of the miR-106a binding site in Angpt1 3' UTR using Targetscan analysis. **(G)** The effect of miR-106a on the luciferase activities of a reporter containing Angpt1 3' UTR in HUVEC culture. **(H)** qPCR analysis of Angpt1 mRNA transcript in HUVEC culture by qRT-PCR analysis. **(I)** Angpt1 secretion levels were assessed in cultured HUVECs by mouse Angpt1 ELISA kit. **(J)** BMMSCs secreted Angpt1 levels were assessed by the mouse Angpt1 ELISA kit. Results were repeated at least three times. All data are expressed as mean±SEM. n=5-8 mice for all groups. *p < 0.05 compared with the WT-Exo and #p < 0.05 compared with the CBS^+/-^-Exo.

After verifying BMMSCs release of exosomes, the present study sought to define the specific exosomal lncRNAs that may regulate the osteogenesis and angiogenesis. We performed a qPCR based lncRNA array (RT2 lncRNA qPCR Array) profile in two representative groups of exosomes: WT-BMMSCs-Exo (WT-Exo) and CBS^+/-^-BMMSCs-Exo (CBS^+/-^-Exo). A total of 26 exosomal lncRNAs were identified as represented in a hierarchical clustering analysis (Figure 3A). Among those, exosomal lnc-H19 was the most robustly downregulated lncRNA in CBS^+/-^-Exo (Figure 3A). A real-time qPCR experiment was subsequently performed to validate the downregulation of lnc-H19 in CBS^+/-^-Exo condition (Figure 3B). Further, the data also confirmed the downregulation of lnc-H19 expression in cultured endothelial cells (HUVEC) treated with CBS^+/-^-Exo compared to HUVEC treated with WT-Exo (Figure 3C). Using lncLocator Database (http://www.csbio.sjtu.edu.cn/bioinf/lncLocator/), the data also found that predicted subcellular location of lnc-H19 in the nucleus (Figure S2A).

### lnc-H19 regulates Angpt1 levels via competitive binding to the miR-106a

Recent studies reported that lncRNAs function as competing endogenous RNAs (ceRNAs) to sponge miRNA targets 29. We attempted to uncover whether any miRNAs could directly target H19. Using a bioinformatics analysis, we examined the seed sequence of miR-106a (which belongs to miR-17-3p family members) in H19 and found that miR‐106a could bind to H19 (Figure 3D). Further, we tested the expression level of miR-106a by using qPCR analysis in the cultured endothelial cells (HUVEC) upon treatment with Exo. The results demonstrate that the transcript level of miR-106a was significantly higher in CBS^+/-^-Exo compared to WT-Exo (Figure 3E). However, the transcript level of other miRNAs (miR-17-3p and miR-20b (miR-17-3p family members)) did not have any significant differences in CBS^+/-^-Exo and WT-Exo groups (Figure S2B-C).

### Angpt1 is a direct target of miR-106a and regulated by lnc-H19

Given that miRNA may target candidate genes by triggering transcriptional inhibition, we used an *in silico* analysis tool, Targetscan. We found that the miR-106 sequence recognized the 23-30 bp of the conserved sequence of Angpt1 (Figure 3F). Luciferase reporter assays and RNA immunoprecipitation (RIP) were performed to confirm the direct binding relationship between lnc-H19 and miR-141. HUVECs were transfected with the Angpt1 3'UTR Lenti-reporter-Luciferase Vector for 48 h. The results showed that Angpt1 luciferase activity was significantly decreased in CBS^+/-^-Exo compared to WT-Exo treated HUVECs (Figure 3G). Further investigation determined whether lnc-H19 was a functional target of miR-106a. We transfected synthetic lnc-H19, or anti-miR-106a, to CBS^+/-^-Exo treated HUVECs and the result suggested that the Angpt1 luciferase activity was significantly improved in CBS^+/-^-Exo+lnc-H19 and CBS^+/-^-Exo+ anti-miR-106a (Figure 3G), thus suggesting that miR-106a did target Angpt1 and induced transcriptional suppression of Angpt1. RNA immunoprecipitation (RIP) assay was also conducted to assess whether the physical interaction of H19 with RISC complex attenuates the function of miR-106a by using a specific antibody against the Ago2 protein. The data found that H19 was preferentially enriched in Ago2-coating beads compared with IgG control (Figure S2D).

Next, we investigate mRNA transcript expression of Angpt1. The qPCR results showed that, Angpt1 mRNA transcript expression was robustly down-regulated in CBS^+/-^-Exo treated HUVECs compared to WT-Exo (Figure 3H). In contrast, Angpt1 secretion was decreased in the culture supernatant of CBS^+/-^-Exo compared to WT-Exo supernatant, as assessed by Mouse Angpt1 ELISA Kit (Figure 3I). Similarly, we also transfected synthetic lnc-H19, or anti-miR-106a, to CBS^+/-^-Exo treated BMMSCs and found that Angpt1 secretion was improved in the culture supernatant of CBS^+/-^-Exo+H19 and CBS^+/-^-Exo+ anti-miR-106a compared to CBS^+/-^-Exo treated BMMSCs (Figure 3J). Collectively, the data showed that lnc-H19 functioned as a molecular sponge to regulate miR-106a expression.

### BMMSC-Exo stimulates angiogenesis *in vitro* and *ex vivo*

**Figure 4:**
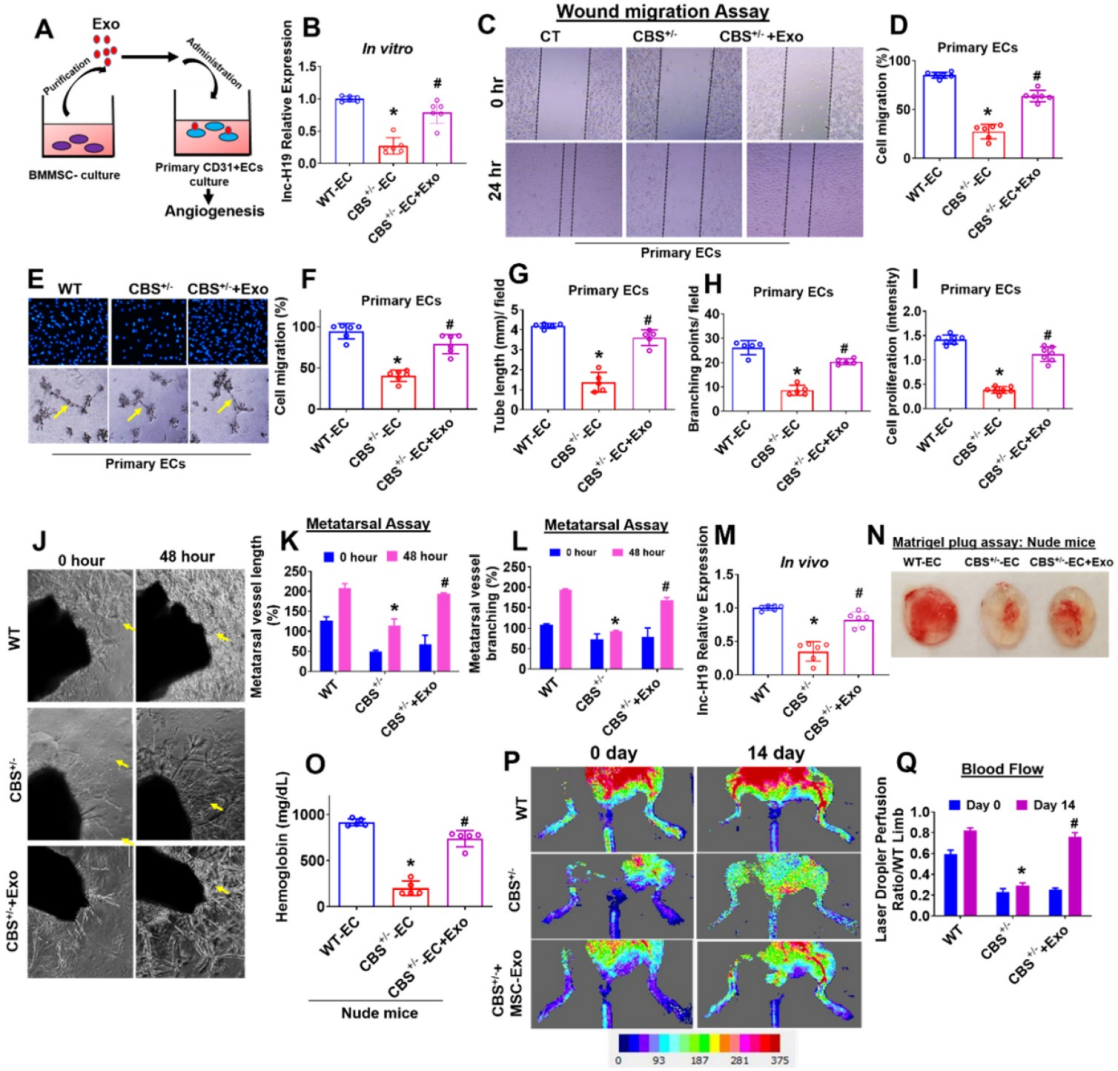
** Angiogenic enhancement by MSCs-Exo *in vitro* and *in vivo.* (A)** Experimental protocol in which primary CD31+ECs derived from both WT and CBS^+/-^ mice and were pre-treated with Exo derived from BMMSCs culture at day 14 and studied angiogenic phenotypes. **(B)** qRT-PCR analysis of lnc-H19 expression in ECs. **(C-D)** Wound healing cell migration study of ECs in the presence of Exo administration. **(E-F)** Trans-well migration (upper) and Matrigel angiogenesis (lower) study of ECS in presence of Exo administration. Migrating cells were represented as the % of cell migration (F). (E) 3D-matrigel tube formation assay of ECs was treated with Exo. The photographs (X20) were taken in five random fields. The scale bar represents 200 µm. **(G-H)** The tube length and branching point/field were quantified in the capillary-like structure by using AngioQuant image software. **(I)** ECs cell proliferation assay in ECs culture treated with Exo administration. **(J-L)** Exo stimulates angiogenesis in mouse metatarsal assays *ex vivo*. The area of metatarsal vessel length and branching points was quantified in vessel-like structures using AngioQuant image software. **(M)** lnc-H19 expression was analyzed in metatarsal tissues *in vivo*. **(N)** Matrigel plug assay shows that Exo is angiogenic in the nude (Foxn1nu/Foxn1nu) mice model *in vivo*. **(O)** Hemoglobin level in Matrigel blood contents were observed using the hemoglobin assay kit. **(P-Q)** Laser Doppler scanning of blood flow over hind limbs on Day 14 after Exo in experimental mice. Results were repeated at least three times. All data are expressed as mean±SEM. n=5-7 mice for all groups. *p < 0.05 compared with the WT-EC and #p < 0.05 compared with the CBS^+/-^-EC.

To investigate the possible role of Exosomes (Exo) in endothelial angiogenesis, primary CD31+endothelial cells (ECs) derived from WT and CBS^+/-^ mice were cultured and treated with BMMSCs Exo (or Exo) (Figure 4A). We tested the lnc-H19 expression level in Exo treated ECs *in vitro*. The data demonstrate that expression level of lnc-H19 was significantly improved in CBS^+/-^-EC+Exo compared to CBS^+/-^-EC condition (Figure 4B). We also examined migration activity by using both a wound healing-migration and a trans-well migration assay. The data showed that Exo treatment improved wound healing-migration in CBS^+/-^-EC+Exo compared to CBS^+/-^-EC (Figure 4C-D). Similar results were observed in the trans-well migration assay (Figure 4E-F). DAPI represents the counterstain for the nucleus in ECs (upper panel). Next, we tested the role of Exo in the formation of 2D-capillary tubes in ECs, another property related to angiogenesis. By using the 2D Matrigel assay, Exo induced the formation of well-organized, capillary-like networks in CBS^+/-^-EC+Exo, compared to those in CBS^+/-^-EC alone (lower panel) (Figure 4F). The tube length and branching points were quantified by AngioQuant software. The data suggests that the area of tube length and branching points were more enhanced in the CBS^+/-^ -EC+Exo condition compared to CBS^+/-^-EC alone (Figure 4G-H). Additionally, we also measured the effect of Exo on ECs proliferation. MTT proliferation assay suggests that cell proliferation activity was indeed increased upon Exo treatment in CBS^+/-^-EC+Exo condition (Figure 4I). Further, we reconfirmed this data with a qPCR analysis. The mRNA transcript of Ki67, a marker of proliferation, was improved in CBS^+/-^-EC+Exo condition compared to CBS^+/-^-EC alone (Figure S3A). Our results suggest that the exosomes significantly promoted angiogenesis in CBS^+/-^ ECs *in vitro*.

Next, we determined the effect of Exo on bone angiogenesis in an *in vivo* model. We performed an *ex vivo* angiogenesis assay using P7 stage mouse metatarsals from WT and CBS^+/-^ mice (the mouse fetal metatarsal provides a unique tool for studying angiogenesis). The data demonstrated that the administration of Exo markedly increased metatarsal angiogenesis in CBS^+/-^-EC+Exo condition as compared with *CBS^+/-^* alone in 48 h (Figure 4J). The quantitative data suggest that Exo enhanced the area of metatarsal vessel length and branching points in CBS^+/-^-EC+Exo condition as compared with *CBS^+/-^* alone (Figure 4K-L). These results demonstrate that Exo mediates angiogenic functions *ex vivo*. Additionally, the data also confirmed that expression level of lnc-H19 was significantly improved in the metatarsal-derived vasculature of CBS^+/-^-EC+Exo compared to CBS^+/-^-EC condition *in vivo* (Figure 4M).

### Effect of BMMSC-Exo on *in vivo* neovascularization and bone blood flow

Next, we used a murine Matrigel plug assay to test the effect of Exo on angiogenesis *in vivo*. In this assay, Matrigel + WT-ECs (as WT-EC), Matrigel + CBS^+/-^ ECs (as CBS^+/-^-EC) and Matrigel + CBS^+/-^EC+Exo (as CBS^+/-^-EC+Exo) were subcutaneously injected into the dorsal region of experimental homozygous nude (Foxn1^nu^/Foxn1^nu^) mice. On the 7th day after the injection, the Matrigel plugs were harvested and examined for neovascularization in the plugs. The data suggest that Exo administration significantly induced more neovascularization in CBS^+/-^-EC+Exo condition (Figure 4N). In contrast, minimal angiogenesis was observed in the *CBS^+/-^*-EC condition (Figure 4N). In addition, Matrigel blood hemoglobin contents were observed in all the experimental conditions in nude mice. The data showed that hemoglobin content was significantly improved in CBS^+/-^-EC+Exo condition as compared to CBS^+/-^-EC, as assessed by a hemoglobin assay kit (Figure 4O). To further determine whether the Exo effect on the bone blood flow in *CBS^+/-^* mice, we examined the blood flow response by using a Laser Doppler imaging flow meter. Results showed significant decline in hind limb blood flow in CBS+/- mice compared to WT mice. Transplantation of Exo resulted in improved hind limb blood flow in CBS^+/-^+Exo condition on day 14 (Figure 4P-Q). Altogether, both *in-vitro* and *in-vivo* data showed BMMSC-Exo promotes angiogenesis (Figure 4).

### BMMSCs-Exo promotes angiogenesis via lnc-H19 dependent Angpt1-NO signaling *in vitro*

**Figure 5:**
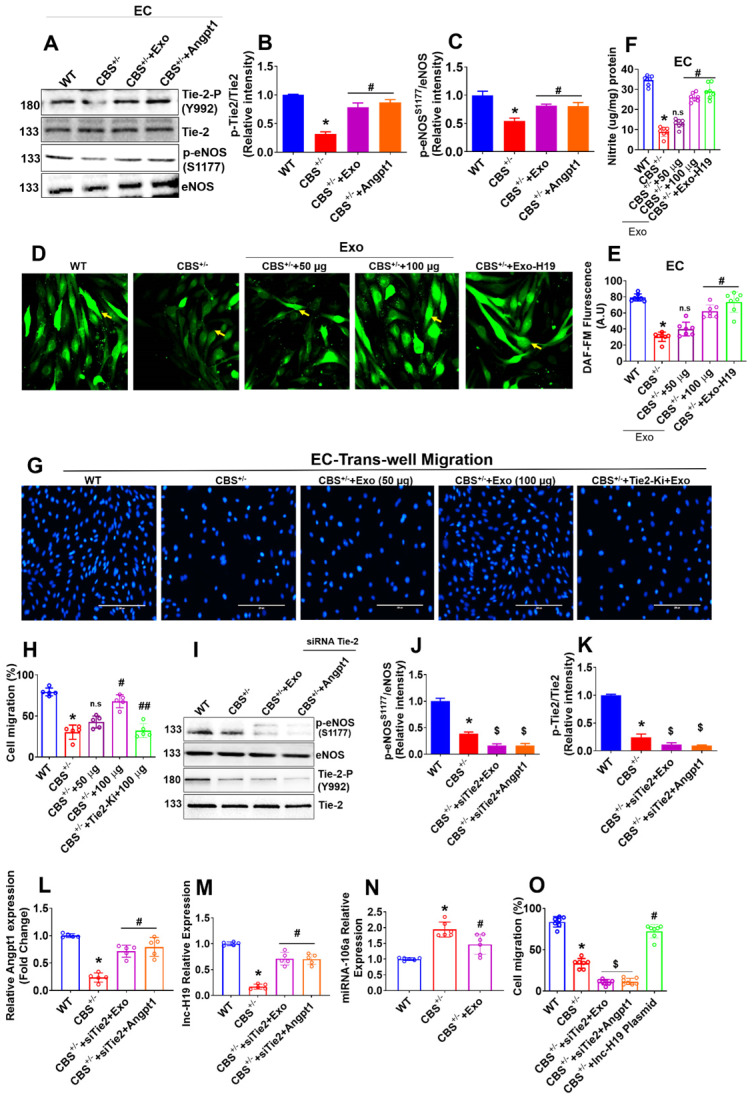
** lnc-H19 mediated Tie2-NO signaling is induced in the promotion of endothelial angiogenesis by Exo. (A-C)** Western blot analysis of Tie2 and eNOS phosphorylation in the ECs. Cells were treated with Exo (100µg), or Angpt1 (100 ng/ml) and phosphorylations of Tie2 (Y992), eNOS (S1179) were analyzed. **(D-F)** ECs were treated with various concentration of Exo (0, 50, 100 µg) or treated with engineered the EXO-H19 for 24h to study endothelial NO and nitrite production by DAF-FM imaging (D-E) and Griess assay method (F). **(G-H)** ECs were treated with Tie2 kinase inhibitor, Tie2-Ki (1.3 nM) and studied ECs migration stimulated by various Exo concentration (0, 50, 100 µg). Photographs (X40) were taken after 24h treatment. The scale bar represents 200µm. **(I-K)** ECs were treated with Exo (100 µg) and Angpt1 in the presence of Tie2 knockdown and studied the phosphorylation of eNOS (S1179) and Tie2 (Y992). **(L-M)** Angpt1 and lnc-H19 expression were analyzed using PCR assay. **(N)** qPCR analysis of miRNA-106 was performed in ECs. **(O)** Trans-well migration activity of ECs in the presence of Exo (100 µg), Angpt1 and overexpression of H19 plasmid. Results were repeated at least three times. All data are expressed as mean±SEM. n=5-7 mice for all groups. *p < 0.05 compared with the WT-EC and #p < 0.05 compared with the CBS^+/-^-EC, ##p < 0.05 compared with the CBS^+/-^-EC+Exo, ^$^p < 0.05 compared with the CBS^+/-^-EC and n.s denotes not significance.

Our results showed that Angpt1 expression and secretion were increased in HUVEC culture following Exo administration via H19 dependent mechanism (Figure 3H-I), which may be involved in endothelial activation and angiogenesis. Therefore, we investigated the molecular mechanisms by which Exo displays angiogenic activity in ECs. A study reported that Tie-2 is a key receptor that induces angiogenesis in endothelial cells (31). Therefore, to dissect the Tie-2 signaling, we administered Angpt1 (100 ng/ml) and Exo independently to CBS^+/-^-ECs and studied the activation of Tie-2. We then studied the activated downstream signaling molecules, such as endothelial nitric oxide synthase (eNOS), nitric oxide (NO) production, associated with endothelial angiogenesis. Importantly, the data demonstrated that Exo or Angpt1 (100 ng/ml) induced the phosphorylation of Tie-2 (Y992) and eNOS (S1179) in CBS^+/-^-EC+Exo or CBS^+/-^-EC+Angpt1 conditions respectively compared to CBS^+/-^-EC alone (Figure 5A-C). Additionally, we have engineered the EXO by loading with lnc-H19 (GFP-tagged) plasmid for delivery into ECs through Exo-Fect transfection method. GFP signal was detected in lnc-H19-GFP-expressing ECs comparing to control empty plasmid using confocal microscopy (Figure S3B). Exo or engineered the EXO-H19 treatment also improved the Tie2 mRNA transcript expression in CBS^+/-^-EC+Exo or CBS^+/-^-EC+Exo-H19 conditions respectively (Figure S3C). Furthermore, intracellular nitric oxide (NO) and NO metabolites (nitrite) were measured using DAF-2DA fluorescence imaging and Griess assay method respectively. The data showed that increased NO and nitrite production was observed in CBS^+/-^-EC+Exo or CBS^+/-^-EC+Exo-H19 (Figure 5D-E and 5F) compared to CBS^+/-^ -EC, respectively. This data suggests that MSC-Exo administration promotes endothelial NO production via the lnc-H19-Tie-2 axis.

To determine the possible crosstalk between Tie-2 signaling and Exo-induced angiogenesis, we examined the effects of Tie2 kinase inhibitor (Tie2-Ki) on Exo stimulated endothelial cell angiogenic properties. The data suggest that pretreatment with Tie2-Ki (100 nM) completely blocked the migration of endothelial cells induced by Exo (Figure 5G-H). The formation of endothelial tube networks induced by Exo was also nearly completely abolished by Tie2-Ki (Figure S4A). To further validate the involvement of Tie-2 signaling in Exo -induced angiogenic activity, we silenced Tie-2 expression with a specific small-interfering RNA (siRNA) in cultured ECs *in vitro*. The efficacy of the siRNA based Tie2 gene knockdown was confirmed by qPCR (Figure S4B). Western blot analysis further revealed that Tie-2 knockdown completely abolished the eNOS and Tie2 phosphorylation in CBS^+/-^-EC+siTie2+Exo condition and CBS^+/^-EC+siTie2+Angpt1 condition (Figure 5I-J, 5I-K). Further, the ligand of the Tie2 receptor, Angpt1 and lnc-H19 expression were tested. The data suggested that Tie-2 knockdown does not induce any changes in the expression of Angpt1 and lnc-H19 expression CBS^+/-^-EC+siTie2+Exo condition and CBS^+/-^-EC+siTie2+Angpt1 condition (Figure 5L-M). Additionally, miR-106 expression was reduced in CBS^+/-^+Exo condition (Figure 5N). Using the Griess assay method, Tie2 gene knockdown completely attenuated the endothelial nitrite production in CBS^+/-^-EC+siTie2+Exo and CBS^+/-^-EC+siTie2+Angpt1 (Figure S4C). Furthermore, Tie2-siRNA completely inhibited ECs migration induced by either Exo or Angpt1 administration, assessed by trans-well migration assay (Figure 5O). These results suggest that Exo might exert angiogenesis via lnc-H19 dependent Angpt1-NO signaling (Figure 5).

### BMMSC-Exo stimulates osteogenesis *in vitro* and *in vivo*

**Figure 6:**
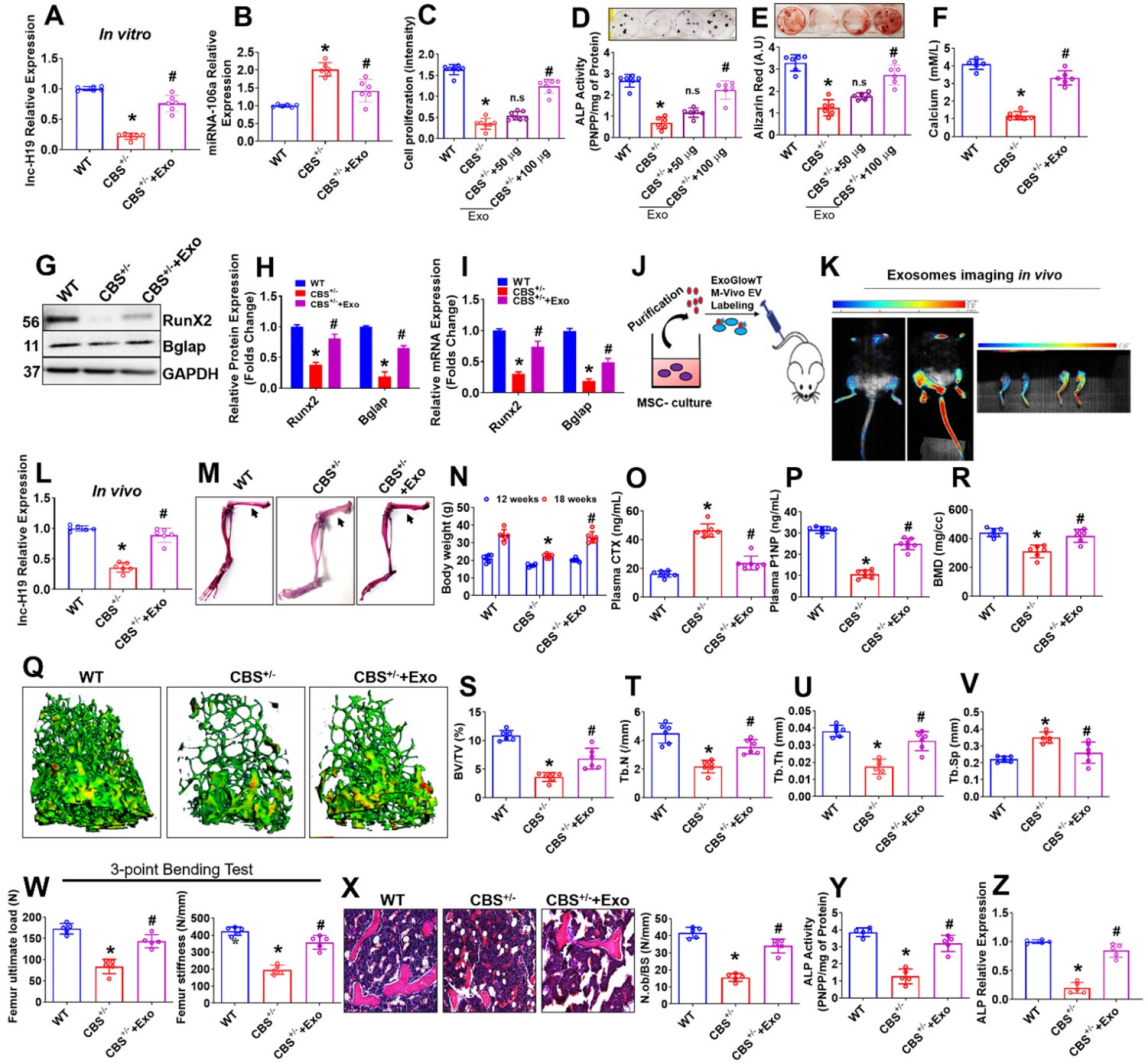
** Exo promotes osteogenesis in the CBS-heterozygous mice *in vitro* and *in vivo.* (A)** lnc-H19 expression in BMMSCs culture using qPCR assay. **(B)** qPCR analysis of miRNA-106 expression in BMMSCs culture. **(C)** BMMSCs were treated with various Exo concentration (0, 50, 100 µg) for 48 h and cell proliferation assay was performed. **(D-E)** BMMSCs were treated with different Exo concentration (0, 50, 100 µg) under osteogenic induction medium (OIM) and ALP and ARS were confirmed on Day 7 and day 21 respectively. **(F)** The bar diagram represents the quantification of cellular calcium was measured in BMMSCs treated with Exo (100 µg) treatment. **(G-H)** Representative western blot analysis for Runx2, Bglap and GAPDH protein control. Densitometry analysis of Runx2 and Bglap protein expression as represented in the bar diagram. **(I)** mRNA transcript expression of osteogenic marker genes (Runx2 and Bglap). **(J-K)** The isolated Exo was i.v. injected via the tail vein of CBS^+/-^ mice at 100µg/mL per mouse for 3 times/week for 8 weeks (a total of 24 injections). Before the last injection, Exo were labeled with the ExoGlow-Vivo dye and administered intravenously via the tail vein into CBS^+/-^ mice. Animals were imaged after 24 h using Biospace Lab Photon Imager* in vivo* and *ex vivo*. **(L)** lnc-H19 expression was analyzed in femoral bone tissue using qPCR assay. **(M)** Alcian Blue and Alizarin Red stained skeletons of a WT and CBS^+/-^ and Exo treated CBS^+/-^ mice (CBS^+/-^+Exo) after 8 weeks of Exo transplantation. **(N)** Bodyweight of experimental mice. **(O-P)** The bar diagram represents the quantification of Plasma CTX (L) and P1NP level using ELISA assay. **(Q)** Representative µCT cross-sectional images of distal femurs in the experimental mice. **(R-V)** The bone phenotype parameters were observed: Bone mineral density (BMD), bone volume per tissue volume (BV/TV) (%), trabecular number (Tb.N) (1/mm), trabecular thickness (Tb.Th.) (mm) and trabecular separation (Tb.Sp.) (mm). **(W)** The graph represents the biomechanical quality of the femurs: ultimate load and stiffness. **(X)** Hematoxylin and eosin (H & E) staining of the trabecular bone volume of the femur. **(Y-Z)** ALP activity and ALP expression was analyzed in femoral bone tissue *in vivo*. Results were repeated at least three times. Data are expressed as mean ± SEM. n = 5-7 mice per group. *p < 0.05 compared with the wild-type (WT) mice, ^#^p < 0.05 compared with the CBS^+/-^ mice. Fig. 6j-z: all the parameters are tested in the experimental mice after 8 weeks of Exo (100 µg) transplantation.

Next, we examined the possible role of Exo in osteogenesis of *CBS^+/-^* mice derived BMMSCs (CBS^+/-^-BMMSC) *in vitro*. First, we successfully observed that there was an improvement in the expression of lnc-H19 in CBS^+/-^+Exo condition compared to CBS^+/-^ condition (Figure 6A). However, the expression of miR-106 was reduced in CBS^+/-^+Exo condition compared to CBS^+/-^ condition (Figure 6B). The data also suggest that the direct application of Exo promoted cell proliferation in CBS^+/-^-BMMSC+Exo condition (Figure 6C). Both ALP activity and bone mineralization (ARS staining) were significantly rescued under Exo treatment in CBS^+/-^-BMMSC+Exo condition at days 6 and 21, respectively (Figure 6D-E). Indeed, the cellular calcium level was significantly increased in CBS^+/-^-BMMSC+Exo *in vitro* (Figure 6F). Sirius red assay also confirmed that collagen production was rescued in CBS^+/-^-BMMSC+Exo (Figure S5A). Both protein and mRNA transcript expression of osteogenic markers, such as Runx2 and Bglap, were increased in CBS^+/-^-BMMSC+Exo compared to CBS^+/-^-BMMSC, respectively (Figure 6G-H, I).

To examine whether Exo transplantation rescues BMMSCs dysfunction and bone loss phenotype in *CBS^+/-^* mice, Exo were labeled with an ExoGlowTM-Vivo EV Labeling Kit (Near IR) and transplanted intravenously via tail-vein route in mice (for 3 times/weeks at 100 µg/mL/mouse/8-weeks -a total of 24 injections) (Figure 6J). Exosome bio-distribution in their bones was assessed 24 h after injection using Biospace Lab Photon Imager* in vivo* and *ex vivo* and compared to controls (Figure 6K). *In vivo* and *ex vivo* fluorescence quantification determined of Exo accumulation in the bone (2.5-fold signal increase in bone tissues) within 24 h after injection (Figure 6K), indicating transplantation influences the biodistribution pattern of exosomes. Indeed, expression level of lnc-H19 was found to be improved in the femoral bone tissue of CBS^+/-^+Exo mice group compared to CBS^+/-^ mice group *in vivo* (Figure 6L).

After the last transplantation of Exo (a total of 24 injections), the whole mounted skeletons of experimental mice were analyzed by Alcian Blue-Alizarin Red staining (Figure 6M). These investigations revealed that all skeletal elements (especially femoral bones) of CBS^+/-^ mice treated with Exo (CBS^+/-^+Exo) looked normal, however, the length of the femur bones remained unaffected in comparison to CBS^+/-^ mice (Figure 6M, Figure S5B). We also found that the body weight and plasma homocysteine level were restored in CBS^+/-^+Exo condition (Figure 6N, Figure S5C, D) compared to CBS^+/-^ mice. In parallel, we quantified various bone remodeling markers in the blood plasma of experimental mice assessed by ELISA. The plasma concentrations of bone resorption marker (CTx) and bone formation marker (P1NP) were recovered in the CBS^+/-^+Exo condition compared to CBS^+/-^ alone, respectively (Figure 6O-P). 3D reconstructions of microCT scan of bone distal femur also revealed that bone mineral density (BMD), bone volume/trabecular volume (BV/TV), trabecular number (Tb.N) and trabecular thickness (Tb.Th) were significantly improved in *CBS^+/-^* mice following Exo transplantation (Figure 6Q-U). Also, the trabecular separation (Tb.Sp) was indeed reduced in CBS^+/-^+Exo condition in comparison with CBS^+/-^ condition alone (Figure 6V). Moreover, Exo transplantation showed a significant increase in bone mechanical strength (ultimate load and stiffness) of the femur in CBS^+/-^+Exo condition (Figure 6W). Furthermore, 2D histological evaluation of the mouse distal femur confirmed the increase in trabecular bone volume in CBS^+/-^+Exo mice condition in comparison to CBS^+/-^ mice. This indicates the osteoporotic phenotype was recovered *in vivo* (Figure 6X). Further, ALP activity and mRNA expression level of ALP was found to be increased in the femoral bone tissue extract of CBS^+/-^+Exo mice group compared to CBS^+/-^ mice group *in vivo* (Figure 6Y-Z). These results suggest that Exo transplantation can rescue osteogenic deficiency of BMMSCs function and osteoporotic phenotype in *CBS^+/-^* mice (Figure 6).

### BMMSC-Exo stimulated lnc-RNA-H19/Tie2-NO axis is essential for BMMSCs osteogenesis

**Figure 7:**
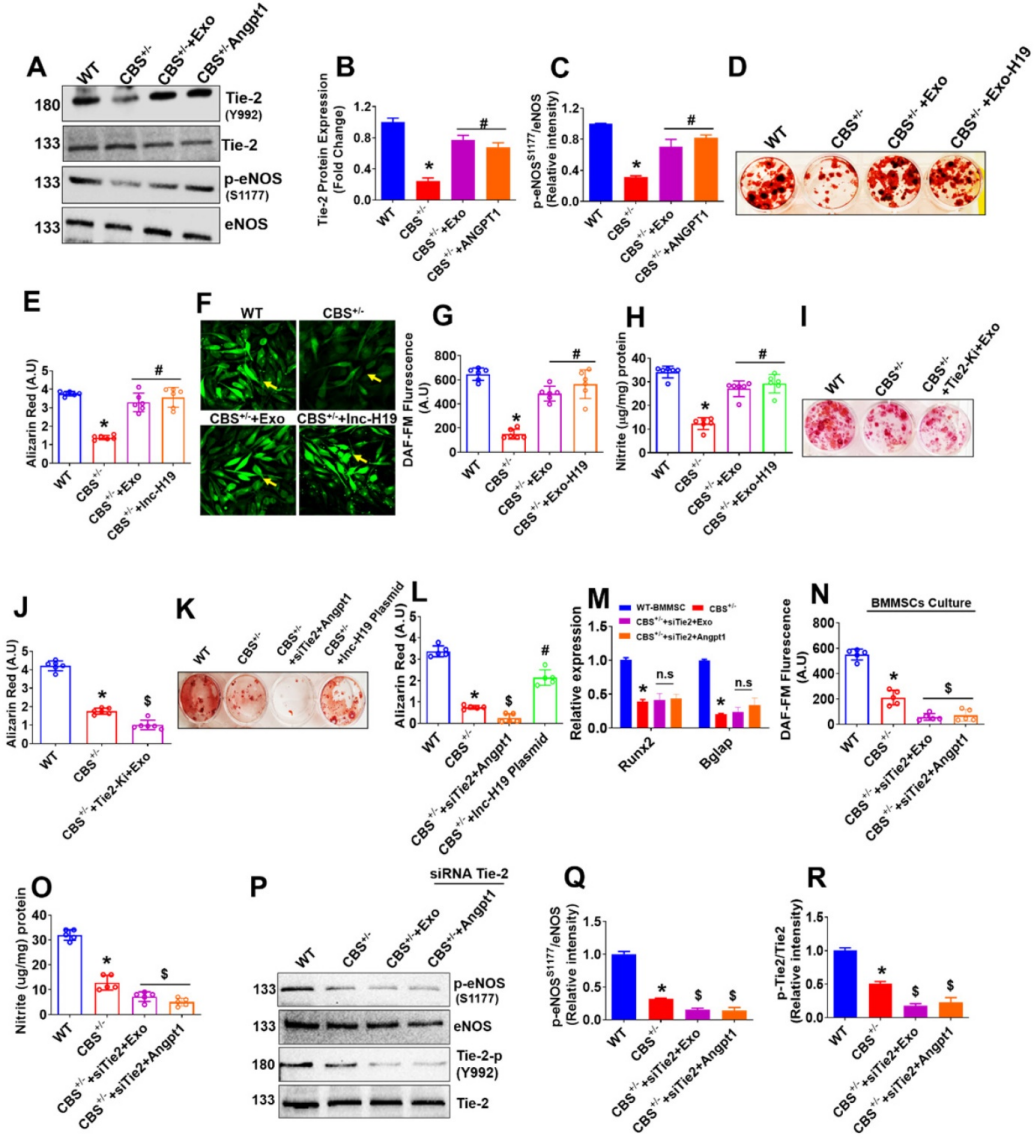
** Exo promotes osteogenesis via lnc-H19 dependent Tie2-NO signaling. (A-C)** Representative western blot analysis of Exo induced phosphorylation of Tie-2 (at the position Y992) (B) and eNOS (at the position S1177) in BMMSCs culture (C). **(D-E)** BMMSCs were treated with engineered the EXO-H19 and Exo (100 µg) and studied its effect on stimulated osteogenesis. ARS staining was performed at 21 days (D). Quantification of the amount of ARS staining (E). **(F-H)** BMMSCs were treated with engineered the EXO-H19. NO and its metabolite nitrite were measured by both DAF-FM imaging (F-G) and Griess method (H). **(I-J)** BMMSCs were treated with Tie-2 inhibitor (1.3nM) and studied osteogenesis and mineralization. **(K-L)** Tie-2 knockdown by siRNA (100ng/mL) and overexpression of lnc-H19 using H19 plasmid in BMMSCs. ARS staining was performed. **(M)** qPCR assay of mRNA transcript expression of osteogenic genes (Runx2 and Bglap) in BMMSCs culture. **(N-O)** NO production and nitrite level was measured in presence or absence of Exo using the DAF-FM fluorimetry and Griess method respectively. **(P-R)** Western blot analysis of phosphorylation of eNOS (S1179) and Tie2 (Y992) in the BMMSCs. Data are expressed as mean ± SEM. n = 5-6 mice per group. *p < 0.05 compared with the WT-BMMSC, #p < 0.05 compared with the CBS^+/-^-BMMSC, ^$^p < 0.05 compared with the CBS^+/-^-BMMSC. Fig. 7a-r: Exo were treated at the concentration of 100 µg to obtain the data from an *in vitro* culture experiment.

Exo is involved in BMMSCs mineralization and bone formation *in-vitro* and *in-vivo*. Therefore, we further examined the mechanistic basis of Exo mediated BMMSCs osteogenesis. We administered Exo and Angpt1 (100 ng/ml) independently to osteogenic BMMSCs culture derived from CBS^+/-^ mice (CBS^+/-^-BMMSC) and studied activation and phosphorylation of Tie-2 and eNOS. Interestingly, the data demonstrated that phosphorylation of Tie-2 (Y992) and eNOS (S1179) protein was increased in the CBS^+/-^+Exo and CBS^+/-^ +Angpt1 condition (Figure 7A-C). Additionally, we have also tested the effect of engineered the EXO-H19 treatment in BMMSCs culture. The data demonstrate that Tie2 and lnc-H19 transcript expression was restored to normal upon engineered the EXO-H19 treatment in CBS^+/-^+Exo-H19 condition respectively (Figure S5E-F). Further, the data also demonstrate that BMMSCs mineralization was indeed improved following Exo treatment or engineered the EXO-H19 treatment in CBS^+/-^-BMMSC (Figure 7D-E). The data also showed that increased NO and nitrite production in CBS^+/-^-BMMSC+Exo or CBS^+/-^-BMMSC+Exo-H19 condition compared to CBS^+/-^ alone (Figure 7F-G, H), respectively.

We examined the effects of Tie2 kinase inhibitor (Tie2-Ki) on BMMSCs osteogenesis to determine the possible cross-talk between Tie-2 signaling and Exo-induced osteogenesis and bone mineralization. The data suggest that pretreatment with Tie2-Ki (100 nM) completely blocked the BMMSCs bone mineralization induced by MSCs-Exo (Figure 7I-J). To further confirm the involvement of the Angpt1-Tie-2 axis in Exo induced osteogenesis and mineralization, we applied a specific small-interfering RNA (siRNA) against Tie-2 gene in cultured BMMSCs *in vitro*. We found that Tie2 siRNA was able to substantially prevent osteogenic differentiation and mineralization of BMMSCs (Figure 7K-L). In addition, the enhanced expression of RUNX2 and Bglap, stimulated by Exo or Angpt1, was significantly suppressed in the presence of Tie-2 knockdown (Figure 7M). The data also suggests that Tie2 gene knockdown completely attenuated the endogenous NO and nitrite production in BMMSCs culture of CBS^+/-^-BMMSC+siTie2+Exo condition and CBS^+/-^-BMMSC+siTie2+Angpt1 condition (Figure 7N-O). To further demonstrate the involvement of Tie-2 signaling in osteogenic activity under Exo treatment, we knocked down Tie-2 expression with a siRNA in BMMSCs culture *in vitro*. Western blot analysis showed that Tie-2 silencing completely abolished the eNOS and Tie2 phosphorylation in CBS^+/-^+siTie2+Exo condition and CBS^+/^+siTie2+Angpt1 condition (Figure 7P-Q, 7P-R). Furthermore, the data showed that Tie-2 knockdown does not cause any changes in the expression of Angpt1 and lnc-H19 expression CBS^+/-^-BMMSC+siTie2+Exo condition and CBS^+/-^-BMMSC+siTie2+Angpt1 condition (Figure S5G-H). Altogether, our results supported Exo treatment is sufficient to stimulate osteogenesis of *CBS^+/-^*-BMMSCs *in vitro* via upregulation of lnc-H19/ Tie-2-NO signaling.

## Discussion

In the past decades, mesenchymal stem cells' therapeutic potential continues to receive widespread attention and has been reported to promote bone regeneration and fracture healing 13. Therefore, we focused on the efficacy of BMMSC-derived exosomes (BMMSC-Exo) secreted during culture conditions. In the present study, we demonstrated that BMMSC-Exo effectively stimulated the BMMSCs osteogenic differentiation and ECs angiogenesis in CBS^+/-^mice. Further, we also demonstrated that BMMSC-Exo is enriched in lncRNA-19 and mediates communication between BMMSCs and ECs during bone homeostasis via lnc-H19-Angpt1/Tie2 signaling in CBS^+/-^ mice model.

Bone formation is a highly complex process that requires close interactions among diverse cell-types 32. Skeletal vascularization and its interaction between bone forming BMMSCs are of essential for repairing skeletal loss 32. This inter-cellular crosstalk between BMMSCs and endothelial cells is likely driven by secretory molecules that work through an autocrine and/or paracrine mechanism, but the target molecules' identifies mediate such interaction have not been fully thoroughly studied. In the current study, we characterized expression profiles of lncRNAs in two groups of exosomes (WT-Exo and CBS^+/-^-Exo) during BMMSCs osteogenesis (Figure 3). Here, we identify lnc-H19 as a novel mediator of crosstalk between BMMSCs and ECs. Although the lnc-H19 was identified in the last few decades, the diverse regulatory role of H19 in bone formation remains unstudied. The lnc-H19 gene is derived from the locus H19-Igf2, which is an imprinted domain found on chromosome 7 in mice and on chromosome 11p15.5 in human 33. This domain is found to be linked to various human pathologies, such as the Silver-Russell and Beckwith-Wiedemann syndromes and also involved in myogenic differentiation 34, growth and development 35. H19 expression is also strongly upregulated in a developing embryo, adult skeletal muscle, and heart. Others reported that lnc-H19 was specifically enriched in exosomes secreted by CD90+ stem cells. It modulates endothelial angiogenic phenotype 36. In the current study, we showed that H19 is known to decrease in CBS^+/-^-Exo compared to WT-Exo. On the other hand, the miR-106 transcript level was significant higher in CBS^+/-^-Exo treated condition. This result gives us the impression that CBS deficiency could lead to down regulation of lnc-H19 in BMMSCs-Exo culture. Based on the ceRNA hypothesis, we also revealed a previously undescribed mechanism by which lnc-H19 potentiates Angpt1-Tie2 signaling by serving as a molecular sponge for miR-106 (Figure 3).

The therapeutic effects of exosomes have been studied in various disease models, including stimulating skeletal muscle 27 and bone regeneration 2, 26. Our lab also reported that embryonic stem cells-Exo could restore the neurovascular unit following ischemic stroke in mice 37. In the present study, we demonstrated that Exo efficiently promotes endothelial migration and metatarsal angiogenesis in CBS^+/-^+Exo condition *ex vivo* (Figure 4). Further, *the data also* showed that Exo stimulates increased *in vivo* neovascularization and hind-limb blood flow in CBS^+/-^ condition (Figure 4). Moreover, Exo could effectively stimulate the ALP activity and calcium nodule formation in BMMSC culture (Figure 6). The administration of Exo reversed the bone loss and improved bone mechanical properties (ultimate load and stiffness) in the CBS^+/-^ mice model *in vivo* (Figure 6).

Using *in silico* analysis with the Targetscan program, the data found that miRNA-106 sequence recognized the 23-30 bp of the conserved sequence of Angpt1 (Figure 3). Indeed, *in silico* Targetscan data was further validated by luciferase assay in various experimental condition. Using anti-miR-106a or synthetic lnc-H19 transfection in CBS^+/-^-Exo conditions prevent the suppressive effect of Angpt1 luciferase activity (Figure 3), suggesting that miR-106a did target transcriptional suppression of Angpt1 through H19 dependent action. Angpt1 and its receptor, Tie2, play a critical role in embryonic vascular development and postnatal angiogenesis 31, 38. Similarly, osteoblast-specific over-expression of Angpt1 increases bone formation 39. However, the understanding of Exo mediated lnc-H19 regulated Angpt1 signaling through miRNA-106 sponging in BMMSCs and ECs CBS^+/-^ condition needs to be further investigated.

Here, we showed that Exo administration is sufficient to promote both angiogenesis and osteogenesis *in vitro* and *in vivo* (Figure 4 & 6) and Angpt1 secretion is markedly increased in response to Exo treatment in CBS^+/-^-ECs and CBS^+/-^-BMMSCs (Figure 3). Moreover, the administration of Exo or Angpt1 enhances Angpt1-Tie2/NO signaling by phosphorylation of Tie-2 (Y992) and eNOS (S1179) in CBS^+/-^+Exo or CBS^+/-^+Angpt1 condition, respectively (Figure 5 & 7). The mechanistic study revealed that depletion of Tie2 expression by siRNA-based gene knockdown approach suppressed their phosphorylation of eNOS in endothelium under CBS^+/-^-EC+Exo or CBS^+/-^-EC+Angpt1 condition, respectively (Figure 5). Further, the mechanistic study showed that the transfection of synthetic H19 restored the NO and its metabolite nitrite in ECs and BMMSCs derived from CBS^+/-^ mice (Figure 5 & 7).

In summary, we demonstrated the potential role of Exo in enhancing osteogenesis and angiogenesis in CBS^+/-^ condition *in vitro* and *in vivo.* We also identified a potential ceRNA network by which lnc-H19 acted as a molecular sponge for the endogenous function of miR-106a, of which it negatively modulates Angpt1 expression. Further, Angpt1 administration increased NO production, neovascularization and bone mineralization via Angpt1-Tie2/NO signaling. This data represents the first demonstration of the NO-dependent nature of BMMSCs-Exo induced angiogenesis and osteogenesis in metabolic osteoporosis (CBS^+/^) mice model via lncRNAH19-Angpt1-Tie2/NO axis.

In summary, our results demonstrate that exosomes derived from BMMSC promote osteogenesis and angiogenesis via lncRNAH19-Angpt1-Tie2/NO signaling in the metabolic CBS^+/-^ mouse model. Moreover, the current understanding of the link between exosomal lncRNAs and bone formation in the physiological condition is still mysterious. However, we discovered that depletion of lncH19 in CBS-Exo disrupts osteogenesis and angiogenesis processes during bone formation. Additional experiments are needed to elucidate the role of lncH19 in osteoclastogenesis and underlying bone loss mechanism. Furthermore, the work would also be warranted to study the osteoprotective function of lncH19 in clinical settings.

## Methods

### Animal model

This study was carefully reviewed and approved by the Institutional Animal Care and Use Committee of the University of Louisville and followed the animal care and guidelines of the National Institutes of Health. 12-weeks-old female wild type C57BL/6J and 129P2-*Cbs^tm1Unc^*/J (CBS KO) mice were purchased from the Jackson Laboratory (Bar Harbor, ME, USA). Female immunocompromised mice (athymic nude, Foxn1^nu^) were also purchased from Jackson Laboratory. The mice groups were the following:

- Wild type C57BJ/L6 mice (WT);
- CBS^+/-^ heterozygous mice fed with methionine (*CBS^+/-^*) mice;
- BMMSC-Exo transplanted in *CBS^+/-^*+Met (*CBS^+/-^*+Exo);
- AOAA treated in *CBS^+/-^*+Met (*CBS^+/-^*+ AOAA);
- CBS CRISPR/Cas9 KO Plasmid transduced in WT mice (CBS KO).

### Drug Preparation and Administration *in vivo and in vitro*

AOAA (CBS inhibitor) was dissolved in 1x PBS. CBS+/-mice were treated with AOAA (100 μM/kg/day/i.p), for 12-weeks through the intraperitoneal (i.p) route. Animals of the wild-type (WT) group received 1x PBS vehicle i.p injections. Similarly, BAY 826 (potent Tie2-Kinase inhibitor (1.3 nM) was dissolved in 100 mM DMSO and treated to BMMSCs and ECs culture condition. Biochemical, molecular, and immune-histochemical analyses were done after the above treatment or its vehicle injection in the groups.

### Isolation of Mouse BMMSCs

Mouse BMMSCs were cultured as per our previously published protocol 7. Briefly, bone marrow (BM) cells were flushed out from the bone cavities of femurs of experimental mice by centrifugation (3000 rpm for 10 min) and collected under alpha minimum essential medium (α-MEM; Invitrogen), supplemented with 2% heat-inactivated fetal bovine serum (FBS; ATCC). Cells were seeded at 20×10^5^ into 100mm culture dishes (Thermo Scientific) and incubated at 37 °C in 5% CO_2_. The adherent cells were cultured for 14 days under α-MEM supplemented with 15% FBS, 2 mM L-glutamine (Invitrogen), 100 U/ml penicillin (Invitrogen), 100 mg/ml streptomycin (Invitrogen) and 50 mg/ml Amphotericin B (Invitrogen). Then, BMMSCs were characterized by both immunofluorescence and flow cytometry analysis with specific markers such as CD73 and CD44 (BioLegend's, San Diego, CA). Concentrations and sources of antibodies that were used are listed in Supplementary Table 2.

### Osteogenic Commitment of Chemically Induced BMMSCs

BMMSCs were cultured under osteogenic chemical stimuli media containing 2 mM β-glycero-phosphate (Sigma), 50 µg/mL L-ascorbic acid-2-phosphate and 10 nM dexamethasone (Sigma). Following the osteogenic differentiation of BMMSCs, the CM was harvested at 14 days. Both alkaline phosphatase (ALP) staining, and alizarin red (ARS) staining were performed at 6 and 21 days, as previously described protocol 1, 40. Mineralized nodules were quantified by absorbing at 405 nm. Likewise, for collagen staining, Sirius red assay was performed at 21 days. Calcium levels were measured in cultured BMMSCs using the Calcium Colorimetric Assay kit. Total proteins and RNAs were isolated after 21 days of induction for further experiments.

### Preparation of Conditioned Medium

Mouse BMMSCs derived conditioned medium (CM) were prepared as previously published in the protocol 41, 42. Briefly, the CM was collected after 24 h of serum starvation from the osteogenically induced differentiated BMMSCs (both WT-CM, CBS^+/-^-CM) at day 14. CM was centrifuged for 30 m at 2000 × g to remove the cells and debris and followed by filtered through 0.22-μm filters. Each conditioned medium was concentrated by 10-fold through 3000 molecular weight cutoff Amicon® Ultra-0.5 Centrifugal Filters (Cat. No: UFC5003, MilliporeSigma, USA) according to the manufacturer's instruction and stored at -80 °C until use for the migration assay, cell proliferation assay and *in vitro* tube angiogenesis assay.

### Exosomes isolation, characterization, and treatment

Mouse BMMSCs derived conditioned medium (CM) and exosomes (Exo) isolation were prepared as previously published in the protocol 41, 42. The collected supernatants were used for isolation of exosomes using both ultracentrifugation method and total Exosome isolation reagent kit (Catalog number: 4478359, Invitrogen, USA), according to manufacturer's instructions. Briefly, the CM was well mixed with reagent mixture by vertexing or pipetting up and down and allowed to incubate samples at 4^0^C overnight. After incubation, samples were centrifuged at 10,000 × g for 1 h at 4 °C. Following completion of centrifugation, supernatants was aspirated, and exosomes are retained in the pellets. Further, the pellets were washed with 1X PBS and centrifuged at 100,000 × g for 1 h at 4 °C. The final pellets are dissolved in 1X PBS and stored at 2 °C to 8 °C for up to 1 week, or at ≤20 °C for long-term storage.

To determine the size of BMMSC-Exo (Exo), we performed Zetasizer Nano ZS analysis (Malvern Instruments, UK). The results were studied using NTA 3.0 software. Protein western blot analysis was performed to detect the CD63 and CD9 expression in isolated Exo. Exo proteins were evaluated by Bradford protein assay (Bio-Rad Laboratories). For local injection into an osteoporotic *CBS^+/-^* mice model, Exo (100 µg/mL) was injected into the tail vein of experimental mice in 3 times/week for 8 weeks (a total of 24 injections).

### Engineering the exosomes with H19 plasmid

The purified Exosomes were transfected with H19 (GFP-tagged) plasmid (Cat#MG201007, ORIGENE) using Exo-Fect™ Exosome Transfection Reagent (SBI) according to the manufacturers' instructions and based on previously published protocol (43). Briefly, isolated exosomes were transfected with H19 (GFP-tagged) plasmid (5 µg) ratio, under diluted to 0.5 µl in Exo-Fect Reagent. The plasmid-loaded exosomes were loaded to culture conditions. The cells were then imaged using confocal imaging for GFP protein presence after 48 h.

### Exosome transplantation and imaging *in vivo* by PhotonIMAGER Optima system

For local injection into an osteoporotic CBS^+/-^ mice model, Exo (100 µg/mL) was administered intravenously via the tail vein of experimental mice in 3 times/week for 8 weeks (a total of 24 injections). Before the last injection, Exo were labeled with the ExoGlow-Vivo dye and was administered via the tail vein into CBS^+/-^ mice. Animals were imaged after 24 h of last injection using *the in vivo* PhotonIMAGER Optima system (Biospace Lab) and showed the preferential biodistribution of labeled Exo in the hind limbs of mice.

### Analysis of the skeleton

Adult, female mice (at age 12-weeks old) WT, *CBS ^+/-^* and CBS^+/-^+Exo mice were deskinned and eviscerated, fixed in 95% ethanol for 3 days, and then transferred into acetone for 1 day. Skeletal staining was performed as previously published 44. Briefly, the whole skeleton was stained with a solution of ethanol: acetic acid: H2O (90:5:5) was supplemented with 0.005% alizarin red S (Sigma) and 0.015% alcian blue 8GS (Sigma) for 3 days at 37 °C. Samples were then rinsed in tap water and cleared for 3-5 days in 1% potassium hydroxide. Then, the skeleton was further cleared using 0.8% KOH-20% glycerol for 5-7 days. Samples were then photographed and stored in 100% glycerol solution. For X-ray analysis, experimental mice were anesthetized, and images were taken with In-Vivo MS FX PRO (Bruker BioSpin) (1). For histological analysis, femur bones were fixed with 4% paraformaldehyde overnight and were subsequently decalcified with 10% EDTA (pH 8.0) for 14 days in paraffin. 6µm sections were prepared using a Leica RM2125 RTS Microtome and stained with hematoxylin and eosin (H&E). The images were recorded at the trabecular bone region by Olympus microscopy and analyzed by using NIH Image J. The data were shown as the percentage of trabecular bones per total bone area of a femur.

### Blood flow by Laser Doppler imaging

Hind-limb blood flow was measured using Laser Doppler flowmetry, according to our previously published protocol 45. Hind-limb blood flow was analyzed in experimental mice on days 0 and 14 after Exo transplantation. Following blood flow measurement, the average flux unit values in the experimental subjects were calculated using Moor LDI software and presented in form of laser doppler perfusion ratio per WT limb group.

### Long non-coding RNA (lncRNA) profiling by RT2 lncRNA PCR array

Total RNA from the two representative groups of exosomes, WT-Exo and CBS^+/-^-Exo was isolated using the RNeasy mini kit (Qiagen, CA, USA) according to manufacturer's instructions. cDNA was synthesized from 200 ng RNA using the RT2 PreAMP cDNA synthesis kit (Qiagen). The cDNA samples were amplified for LncRNAs profiling in a 96 well format RT2- lncRNA PCR array (LAMM-001Z, Mouse LncRNA finder, Qiagen) using 2X RT2 SYBR Green PCR kit (Qiagen). The amplification was performed in the LightCycler® 96 Instrument (Roche, Life Science). The significantly upregulated/downregulated lncRNA in WT-Exo vs. CBS^+/-^-Exo were analyzed using Qiagen software and further validated using the relative quantification approach 2-ΔΔCT.

For miRNA detection, the purification of microRNA and cDNA synthesis was carried out using a miRNeasy Mini kit and miScript II RT kit (QIAGEN) respectively. The qRT-PCR was performed to analyze the target miRNA expression using miScript SYBR Green PCR Kit (QIAGEN) in LightCycler® 96 Instrument (Roche, Life Science). miR-106a was normalized with U6. The relative expression level was calculated by normalization with the signal for U6 expression using the 2-ΔΔCT method.

### Gene expression studies using qPCR

Gene expression analysis was performed as previously described 46, 47. Total RNA was isolated with TRIzol® reagent (Invitrogen, Grand Island, NY, USA) from the BMMSCs culture and reverse transcription of cDNA was performed using ImProm-II™ Reverse Transcription System (Promega, USA). Gene amplification was carried out using the PerfeCTa SYBR Green FastMix (Quanta BioSciences) in the LightCycler® 96 Instrument (Roche, Life Science). Relative quantification of PCR products was calculated in the experimental samples according to the loading control mRNA expression of GAPDH. The primer sequences for target gene amplification were listed in Supplementary Table 1.

### *In silico* Analysis

*In silico* analysis, programs were performed to predict potential targets of miR-106a, including Targetscan (http://www.targetscan.org/), and miRcode (http://www.mircode.org/index.php). Besides, we also used DIANA Tools (http://diana.imis.athena-innovation.gr/DianaTools/index.php?r=lncBase/index) to identify predicted direct miRNA for lncRNA-H19 or LncRNAs sponging activity. Using lncLocator: lncRNA subcellular localization predictor software (http://www.csbio.sjtu.edu.cn/bioinf/lncLocator/), we predicted the subcellular localizations of lncRNA-H19.

### ANGPT1-UTR-3' Luciferase assay

ANGPT1 luciferase activity was performed as previously reported with little modifications 48, 49. Angpt1 3'UTR Lenti-reporter-Luciferase Vector was purchased (ABM, Richmont, Canada).The cultured ECs and BMMSCs were pre-transfected with Angpt1 3'UTR Luc Vector and followed by anti-miR-106 (100 ng/mL) or lncRNA-H19 (50 ng/mL) using Lipofectamine 2000 (Invitrogen, Carlsbad, CA, USA) for 48 h. Cell lysates were prepared and luciferase activity was performed using the Dual-Luciferase Reporter Assay (Promega, Madison, WI, USA). Results were normalized to the Renilla luciferase.

### RNA immunoprecipitation (RIP) assay

RNA immunoprecipitation assays were performed as previously described with some modification 50, 51. BMMSCs were transfected with synthetic lncRNA-H19 and following 80-90% confluency, cells were subsequently fixed by 2% formaldehyde. The cell pellets were further suspended in the complete RIP lysis buffer (Millipore). The supernatant from cell lysates was collected using centrifugation at the speed of 14000 rpm. To prepare antibody-coated magnetic beads, Protein G Sepharose 4 was washed with cold NT2 buffer and subsequently incubated with Ago2 antibody or corresponding negative control IgG. To remove the non-specific binding, the Sepharose beads were incubated with 10 mg/ml proteinase K (Sigma-Aldrich, USA) to digest the protein and RNA was isolated by immunoprecipitation. The RNA concentration was determined, and quality tested and then retrieved RNAs were subjected to RT-qPCR assays to evaluate the enrichment degree of H19.

### siRNA delivery and target gene knockdown *in vitro*

To investigate the role of lncRNA-H19 and Tie2 function in both osteogenesis and angiogenesis *in vitro*. siRNAs against Tie2 (100 ng/mL) or negative control, synthetic lncRNA-H19 (50 ng/mL) and antimir-106a (5′-CUACCUGCACUGUAAGCACUUUU-3′ and the miRNA inhibitor negative control: 5′-CAGUACUUUUGUGUAGUACAA-3′) were purchased (Sigma, USA) and were transfected to the cultured ECs and BMMSCs for 48 h using Lipofectamine™ 2000 Transfection Reagent (Invitrogen) according to the manufacturer's instructions.

### Overexpression of lncRNA H19 *in vitro*

To confirm that lncRNA H19 functions as an inducer of osteogenesis and angiogenesis, we transfected BMMSCs (1×10^6^ cells per well) culture with the pcDNa3.1(+)A009-H19 (Plasmid #122473, addgene) plasmid to overexpress H19 (lncRNA H19 group). Briefly, cells were allowed to be grown to 90% confluence in 6-well plates and the transfected pcDNa3.1(+)_A009-H19 and negative control plasmid using Lipofectamine 2000 (cat. No: 11668030, Life Technologies Corporation, USA) according to the manufacturer's instructions. After 48 h of post-transfection, cells were refreshed with cultured medium and allowed to grown on osteogenic medium for 2-weeks. At the end of culture, exosomes were purified from the collected CM under serum free condition at day 14 and was proceeded for downstream experiments such as osteogenic and angiogenesis assay.

### Generation of CBS KO BMMSCs Using CRISPR/Cas9 Gene-editing Technology

CBS-deficient BMMSCs (CBS-KO) were generated as our previously described protocol 7. Briefly, BM cells were isolated and cultured under osteogenic medium (α-MEM supplemented with 15% FBS, containing 2 mM β-glycero-phosphate, 50 µg/mL L-ascorbic acid-2-phosphate and 10 nM dexamethasone) for 2-weeks. The differentiated BMMSCs were transfected with CRISPR/Cas9 KO Plasmid (Santacruz Biotechnology, sc-400877, CA, USA), by using Lipofectamine™ 2000 Transfection Reagent (Invitrogen). Then cells were incubated for 24-h under the serum-deprived condition at 37 °C. After incubation, cells were cultured additional 24-h and CRISPR/Cas9 KO Plasmid was visually confirmed by detection of the green fluorescent protein (GFP) via confocal microscopy.

The adherent cells were cultured for 14 days under α-MEM supplemented with 15% FBS, 2 mM L-glutamine (Invitrogen), 100 U/ml penicillin (Invitrogen), 100 mg/ml streptomycin (Invitrogen) and 50 mg/ml Amphotericin B (Invitrogen).

### Immunoblotting

Western blot analysis was performed as previously described 52, 53, 54.Protein lysates/extracts (40 μg) were prepared from the BMMSCs, ECs, and exosomes using RIPA lysis buffer (Thermo Fisher Scientific) and loaded on a sodium dodecyl sulfate-polyacrylamide gel (SDS-PAGE) independently. Separated proteins in the gels were transferred to polyvinylidene difluoride (PVD) membranes using an electrotransfer apparatus (Bio-Rad, USA). Following blocking the membrane with 5 % non-fat dry milk (1 h), the membranes were probed with a primary antibody (anti-RUNX2 (ab23981), anti-Bglap (ab13420), anti-Tie-2 (ab111074) and anti-phosphor Tie2 (Y992, ab151704), anti-NOS3 (ab76198) and anti-phosphor NOS3 (S1177, ab230158), anti-CD9 (ab223052), anti-CD63 (ab8219)) for 2 h at 4 °C. Then membranes were further incubated with a secondary antibody conjugated with horseradish peroxidase for 1 h at room temperature. The membranes were developed with the ECL Western blotting detection system (GE Healthcare, Piscataway, NJ, USA) and the image was recorded using a gel documentation system (Bio-Rad, USA). Each target protein band density was normalized with a respective GAPDH density using Image Lab densitometry software (Bio-Rad) and expressed as fold changes to control.

### Flow cytometry analysis

Surface antigens of BMMSCs were analyzed using flow cytometry study according to our previously published protocol 1. Briefly, 1.0 ×10^5^ BMMSCs were stained with 5 µl of each FITC-conjugated CD73 antibodies and APC-conjugated CD44antibodies or isotype-matched control IgGs (Biolegends, USA) at 4 °C for 45 min. The samples were fixed with 2% paraformaldehyde for 30 min and analyzed by BD Accuri™ C6 Cytometer (BD Bioscience, San Jose, CA). Nonspecific fluorescence was determined with isotype-matched control antibodies (Biolegends, USA).

### Immunofluorescence staining and confocal imaging

BMMSCs were seeded on 8-well chambered slides (Nunc® Lab-Tek™, Thermo Scientific). Following confluence, cells were fixed with 4% PFA. The cells were further incubated with FITC-conjugated CD73 antibodies and APC-conjugated CD44 antibodies (1:100) at 4 °C overnight. Then cells were counterstained with 4',6-diamidino2-phenylindole (DAPI) for 20 min. The fluorescence images were photographed using a confocal scanning microscope. Concentrations and sources of antibodies that were used are listed in Supplementary Table 2.

### Cell proliferation assay

The proliferation of both BMMSCs and endothelial cells was performed by MTT (3-(4, 5-Dimethylthiazol-2-yl)-2, 5-Diphenyltetrazolium Bromide) assay as previously described 55. Briefly, 1×10^3^ cells were seeded on 96-well plates and allowed to culture for 1-2 days in the presence or absence of Exo. The cultures were incubated with MTT solution (1 mg/ml) (Sigma) for 4 h until the purple precipitate is visible. Then 100 µl dimethyl sulfoxide (DMSO) was added to the precipitate and allowed to leave at the room temperature in the dark for 2 h. The absorbance was recorded at 570 nm. The MTT assay was repeated for 3 independent samples for each experimental group.

### Trans-well migration and wound healing migration assay

Transwell migration assay was performed to study the endothelial migration using 24-well Transwell plates containing 8 μm pore size polycarbonate filters. Briefly, the cultured CD31+ECs were trypsinized and seeded at the density 5x10^3^ cells in the upper well. The serum-free α-MEM was supplemented with Exo was added to the lower chamber of the Tran-well system. After 24 h of incubations, migrated endothelial cells were fixed with 4% PFA and stained with a nuclear stain DAPI solution for 20 min at room temperature. Images were acquired in five randomly chosen fields/well using a confocal laser scanning microscope and counted. Cell migration was expressed as % of control.

For wound migration assay, CD31+ECs (1×10^6^ cells/well) were cultured in 24-well culture plates. Following confluence, a scratch wound was created with a 200 µl pipette tip. The cells were washed with PBS and supplemented with serum-free DMEM plus Exo. After 24h of incubation, migrated cells were photographed and quantified using the Image J software program (National Institute of Health, Bethesda, MD, USA).

### Matrigel 3D- angiogenesis

Tube formation assays were performed as previously described 56. At subconfluence, ECs were incubated with 1,1'-dioctadecyl-3,3,3',3'-tetramethylindocarbocyanine-labeled acetylated low-density lipoprotein (DiI-acLDL, Molecular Probes, USA) at 10 µg/ml for 2 h. Then, cells were seeded at the density 0.5×10^5^ cells per well into 96-well plates pre-coated with 50µL of growth factor-reduced Matrigel (354230, Invitrogen, USA) and allowed to incubate for 8 h at 37 °C in serum-free DMEM in the presence/absence of Exo as indicated. The endothelial networks appeared under matrigel support and five random areas for each sample were selected and imaged using a fluorescence/bright field microscope. Using the AngioQuant software, total tube length and branch points were measured in each well.

### Measurement of NO production

Intracellular NO and nitrite production were measured as previously described 57. Intracellular NO production was monitored using fluorescent NO indicator Diaminofluorescein-FM (DAF-FM, Sigma). Cultured ECs and BMMSCs were incubated with DAF-FM (10 µM) dissolved in PBS for 30 min at room temperature. Following incubation, excess DAF-FM probes were washed with PBS. The fluorescence images were taken using a confocal laser scanning microscope. The amount of NO was also evaluated by measuring the fluorescence intensity excited at 495 nm and emitted at 515 nm in the fluorimeter. Similarly, NO metabolite nitrite in cultured ECs and BMMSCs were measured by colorimetric Griess assay in a 96-well plate. Briefly, 150 μl of the cell supernatant was mixed with the same volume of Griess reagents (58 mM sulphanilamide in 2.5% phosphoric acid plus 12 mM N-(1-naphthyl)ethylenediamine in 2.5% phosphoric acid) were added and the samples were incubated for 25 min in the dark. The absorbance was read at 570 nm using a microplate reader. Nitrite levels were calculated using standards of sodium nitrite in a range of 10-100 μg were used for calibration of the assay.

### *Ex vivo* metatarsal angiogenic assay

P7 stage mouse metatarsals from WT and *CBS^+/-^*mice were dissected as described earlier 58. Five bone explants per experimental group were transferred to 24-well tissue culture plates pre-coated with Matrigel matrix, containing DMEM (ATCC) with 10% FBS and penicillin/streptomycin (PS), and allowed to culture for 7 days. The medium was replaced in every alternative day under the presence/absence of Exo. After 9 days of culture, bone explants were fixed and blood vessel formation was visualized by bright-field microscopy. Area of metatarsal vessel length and branching points were calculated using AngioQuant software.

### *In vivo* Matrigel plug assay

The *in vivo* Matrigel plug assay was performed as previously described 59. 5-weeks-old female immunocompromised Nude mice (homozygous for Foxn1^nu^ (Nu/J), JAX stock #002019) were injected subcutaneously near their abdomen midline with 0.5 ml of Matrigel plus WT-ECs (as WT-EC) or Matrigel plus CBS^+/-^ ECs (CBS^+/-^ EC) or Matrigel plus CBS^+/-^EC+Exo (CBS^+/-^-EC+Exo) to promote angiogenesis. Each mouse received two plugs and subtotal 5 mice were used for each experimental group. After 7-days of implantation, the mice were euthanized and Matrigel plugs were removed and photographed. To quantitate the formation of functional blood vessels, Drabkin hemoglobin (Hb) assay was performed to measure the amount of Hb. On the other hand, the plugs were then fixed in 4% paraformaldehyde and bright-field images were visualized using an Olympus microscope.

### Mouse angiopoietin 1 ELISA Kit

ELISA assay was used to determine the amount of angiopoietin 1 (Angpt1) in the BMMSC and endothelial culture supernatants of the experimental mice. Both BMMSC-derived Angpt1 and endothelial supernatants Angpt1 were measured using a commercial Mouse angiopoietin 1 ELISA Kit (Catalog# MBS704424) from MyBioSource, as per the manufacturer's instructions. Briefly, 100 μL of sample or standards in Reagent Diluent, was added to each well of the provided plate and allowed to incubate at room temperature. Following washing of each well, 100 μL of the detection antibody was added and incubated at the room temperature for 2 h. Then, 100 μL of the working dilution of Streptavidin-HRP to each well was loaded and kept in the dark room for 20 min and followed by addition of 100 μL of Substrate Solution to each well. After 20 min of reactions, 50 μL of Stop Solution was added to each well and optical density was determined using a microplate reader at 450 nm. The unknown concentration of Angpt1 in the samples was calculated from using standard concentration of Angpt1.

### Bone analyses by micro-computed tomography (microCT)

Micro-computed tomography (μCT) imaging of the femur was performed as our previously described protocol 60. Distal femoral metaphyses were scanned on a high-resolution CT scanner (Skyscan 1174). The bone samples were fixed overnight with 4% paraformaldehyde followed by stored with 70% ethanol until usage. After scanning, 3D-reconstruction of the femur was carried out using SkyScan Nrecon software. Trabecular bone architectural properties were studied. Trabecular bone volume relative to tissue volume (BV/TV, %), trabecular thickness (Tb.Th), trabecular number (Tb.N) and trabecular separation (Tb.Sp) were determined for a standardized region in the distal femoral metaphysis.

### Bone mechanical testing by 3-point bending test

Bone mechanical testing of femurs was performed as our previously described protocol 1. Bone femurs were fixed in 4% PFA for overnight and stored in 70% ethanol at -80 °C until usage. A 3-point bending test was performed to study bone mechanical properties of cortical strength. All femur bones were tested under a load applied at a constant rate of 20 mm/min. This test predominantly measured the cortical bone strength parameters like maximum load (ultimate strength) and stiffness (elastic deformation) by using Bone Strength Tester TK-252C (Muromachi Kikai Co. Ltd., Tokyo, Japan).

### Statistical Analysis

Data analyses and graphical presentations were performed with GraphPad Prism, version 8.3.0 (GraphPad Software, Inc., La Jolla, CA). Data are represented as the mean value ± standard error of the mean (SEM). The experimental groups were compared by one-way analysis of variance (ANOVA) in combination with Tukey's multiple comparison tests. The significance of differences between the two groups was determined using Two-tailed, unpaired Student's *t*-test. *p* < 0.05 was considered statistically significant. All of the experiments presented in the present study were repeated at least three times. Appropriate sample sizes were calculated to ensure statistical significance could be determined.

## Supplementary Material

Supplementary figures and tables.
