# Supplementary material for: Exosomal lncRNA-H19 promotes osteogenesis and angiogenesis through mediating Angpt1/Tie2-NO signaling in CBS-heterozygous mice
Source: Theranostics. 2021 Jun 22;11(16):7715–34. doi: 10.7150/thno.58410 (PMC8315071; doi:10.7150/thno.58410)
Supplement: Supplementary file 1 — Supplementary figures and tables. [file thnov11p7715s1.pdf]

# Supplemental Information

## Exosomal lncRNA-H19 promotes osteogenesis and angiogenesis through mediating Angpt1/Tie2-NO signaling in CBS- heterozygous mice

Jyotirmaya Behera[1], Anil Kumar[2], Michael J. Voor[3], Neetu Tyagi[1]*

[1]Bone Biology Laboratory, Department of Physiology, School of Medicine, University of Louisville, Louisville, KY 40292, USA.

[2]James Graham Brown Cancer Center, Department of Microbiology & Immunology, University of Louisville, KY 40202, USA.

[3]Departments of Orthopaedic Surgery and Bioengineering, School of Medicine and Speed School of Engineering, University of Louisville, Louisville, KY 40292, USA.

**Running title:** Role of the exosome in bone formation and vascularization in CBS-heterozygous mice.

**Address for Correspondence:**

Neetu Tyagi, Ph.D., FAPS
Associate Professor
Department of Physiology
Health Sciences Center, A-1201,
University of Louisville
Louisville, KY 40202
Phone: 502-852-4145
Fax: 502-852-6239
E-mail: n0tyag01@louisville.edu

**Supplementary Figure S1.**

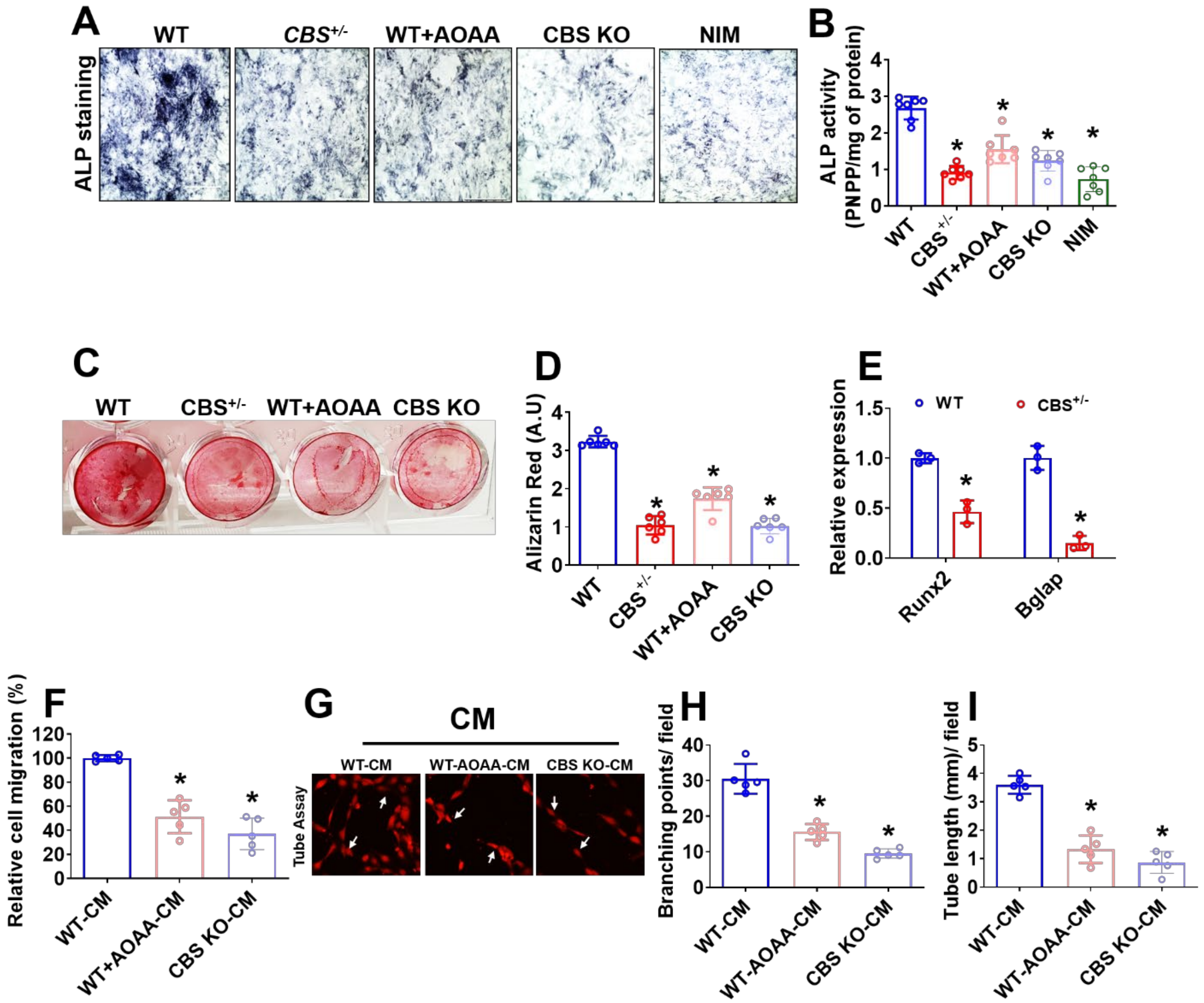


**Supplementary Figure S1. Osteogenesis of BMMSCs in the different experimental mice.** (A-D) BMMSCs were cultured under osteogenic induction medium (OIM) and non-induction medium (NIM) in different experimental conditions. Osteogenic differentiation was confirmed by both ALP on Day 7 (A-B) and ARS staining on day 21 (C-D) respectively. The scale bar represents 200 µm. (E) Expression profiles of the osteogenic markers, Runx2, and Bglap were examined by qPCR analysis. (F) ECs migration was tested using Trans-well migration assay. (G-I) 3D-matrigel tube formation assay of ECs was performed. The photographs (X20) were taken in five random fields. The scale bar represents 200 µm. The tube length and branching point/field were quantified by using AngioQuant image software. Results were repeated at least three times. All data are expressed as mean±SEM. n=5-7 mice for all groups. *p< 0.05 compared with the WT.

**Supplementary Figure S2.**

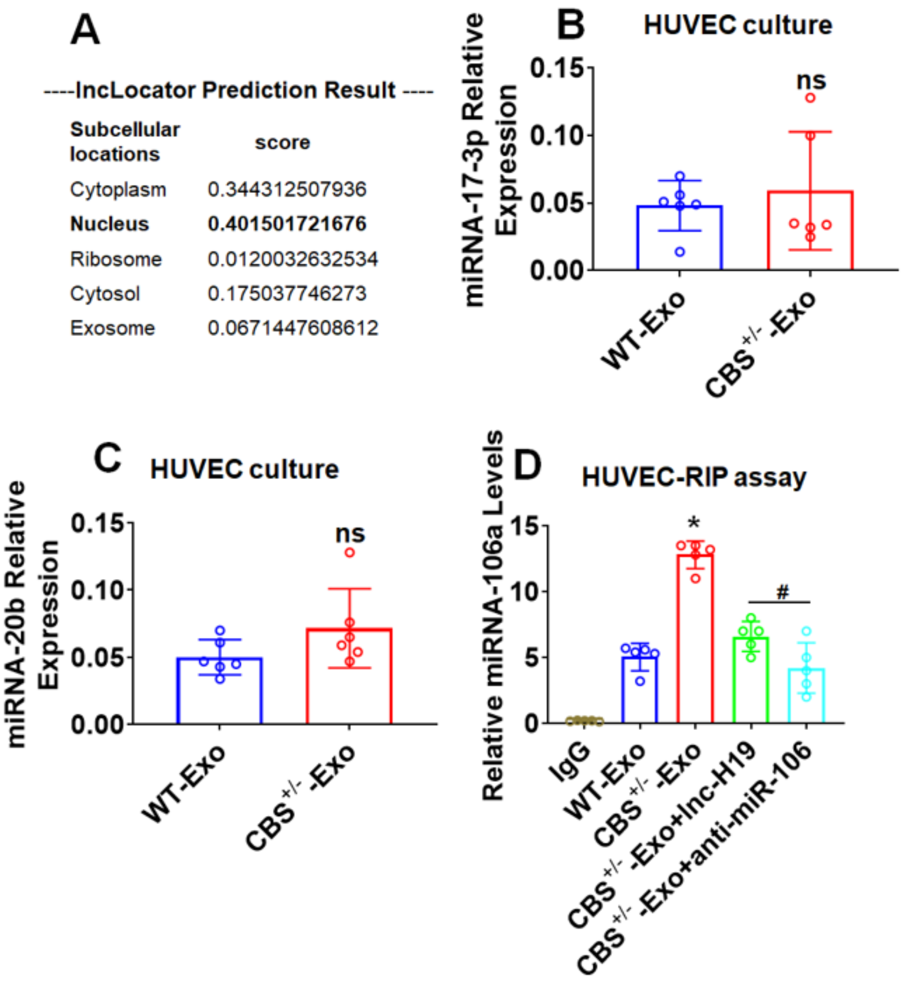


**Supplementary Figure S2. miRNA transcript expression of miR-17-3p family members and RNA immunoprecipitation in HUVEC culture.** (A) Prediction of lncRNAH19 cellular location using lncLocator Database (http://www.csbio.sjtu.edu.cn/bioinf/ lncLocator/) (B-C) miR-17-3p family members: miR-17-3p and miR-20b were analyzed in HUVEC culture under $CBS^{+/-}$-Exo treatment by qPCR. (D) HUVEC culture was transfected with synthetic lncH19 or anti-miR-106a before the Exo treatment. RNA immunoprecipitation assay was performed to assess the physical interaction of H19 with the RISC complex by using a specific antibody against Ago2 protein. Results were repeated at least three times. All data are expressed as mean±SEM. n=5-6 mice for all groups. *$p < 0.05$ compared with the WT-Exo and #$p < 0.05$ compared with the $CBS^{+/-}$-Exo.

**Supplementary Figure S3.**

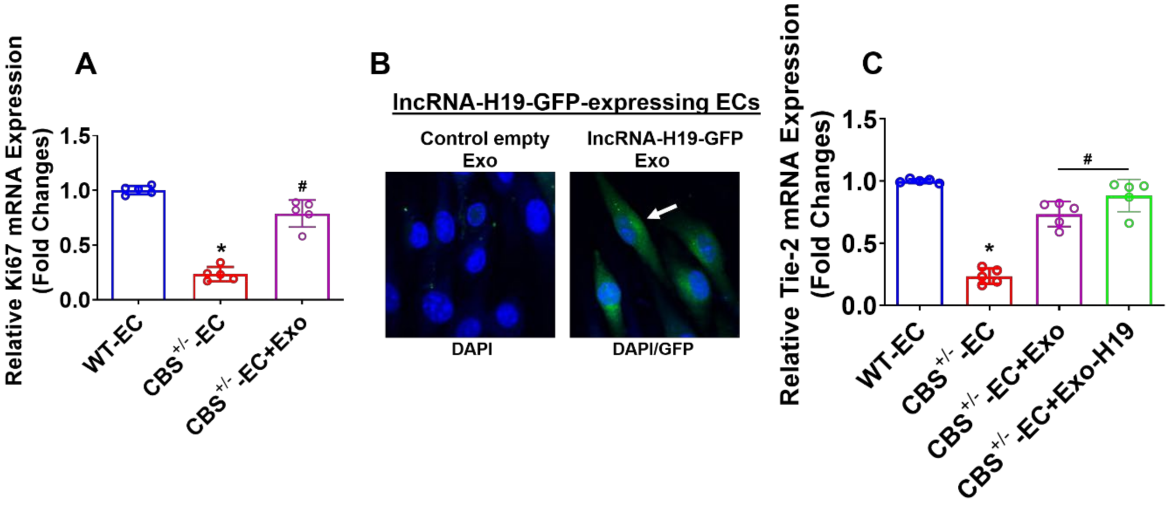


**Supplementary Figure S3. mRNA transcript expression of proliferation marker Ki67 and effect of engineered Exo with lnc-H19 on Tie-2 expression in the endothelium.** (A) Ki67 mRNA transcript expression by qPCR analysis. (B) We used Exo-Fect to transfect 5 µg of H19 (GFP-tagged) plasmid DNA into exosomes, and then added the plasmid-loaded exosomes to ECs. The cells were imaged after 48 h for detection of GFP protein signal. The appearance of GFP signal (plasmid cargo, white arrow) in ECs indicates successful cargo delivery. (C) Tie-2 mRNA transcript expression in ECs qPCR analysis. Results were repeated at least three times. All data are expressed as mean±SEM. n=5 mice for all groups. *$p < 0.05$ compared with the WT-EC and #$p < 0.05$ compared with the $CBS^{+/-}$-EC.

**Supplementary Figure S4.**

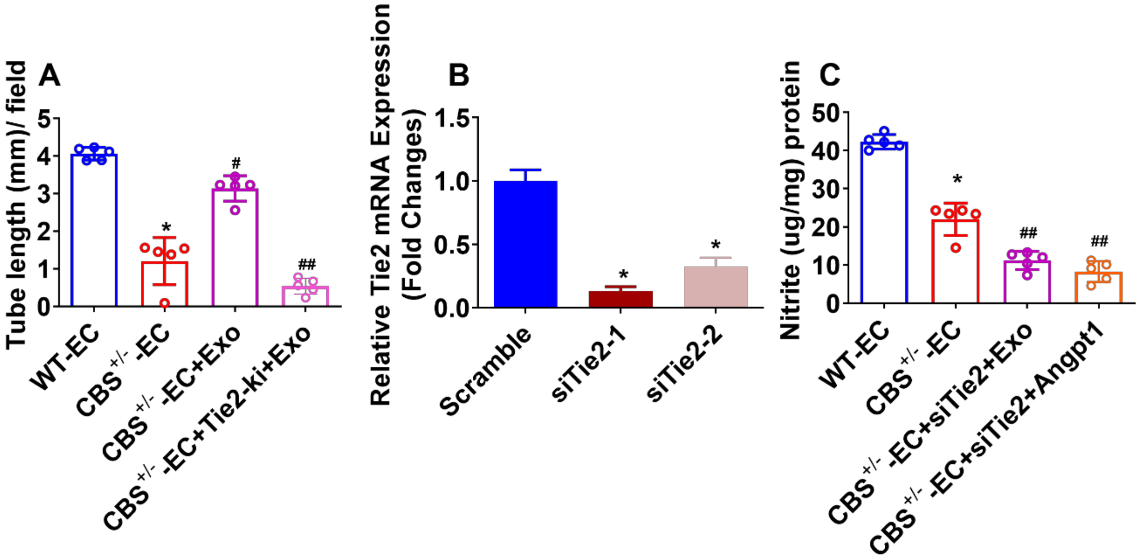


**Supplementary Figure S4. The inhibition of Tie-2 expression reduces angiogenesis and NO metabolite level in the endothelium. (A)** The effect of Tie-2 inhibition by potent Tie-2 inhibitor (1.3nM) reduces 3D-Matrigel angiogenesis. **(B)** qPCR assay was performed to confirm the efficacy of the siRNA1 and siRNA2 based Tie2 gene knockdown in endothelial culture. **(C)** NO metabolite nitrite was measured using the Griess assay method. Results were repeated at least three times. All data are expressed as mean±SEM. n=5 mice for all groups. *p< 0.05 compared with the WT-EC, #p< 0.05 compared with the $CBS^{+/-}$-EC, and ##p< 0.05 compared with the $CBS^{+/-}$-EC+Exo.

**Supplementary Figure S5.**

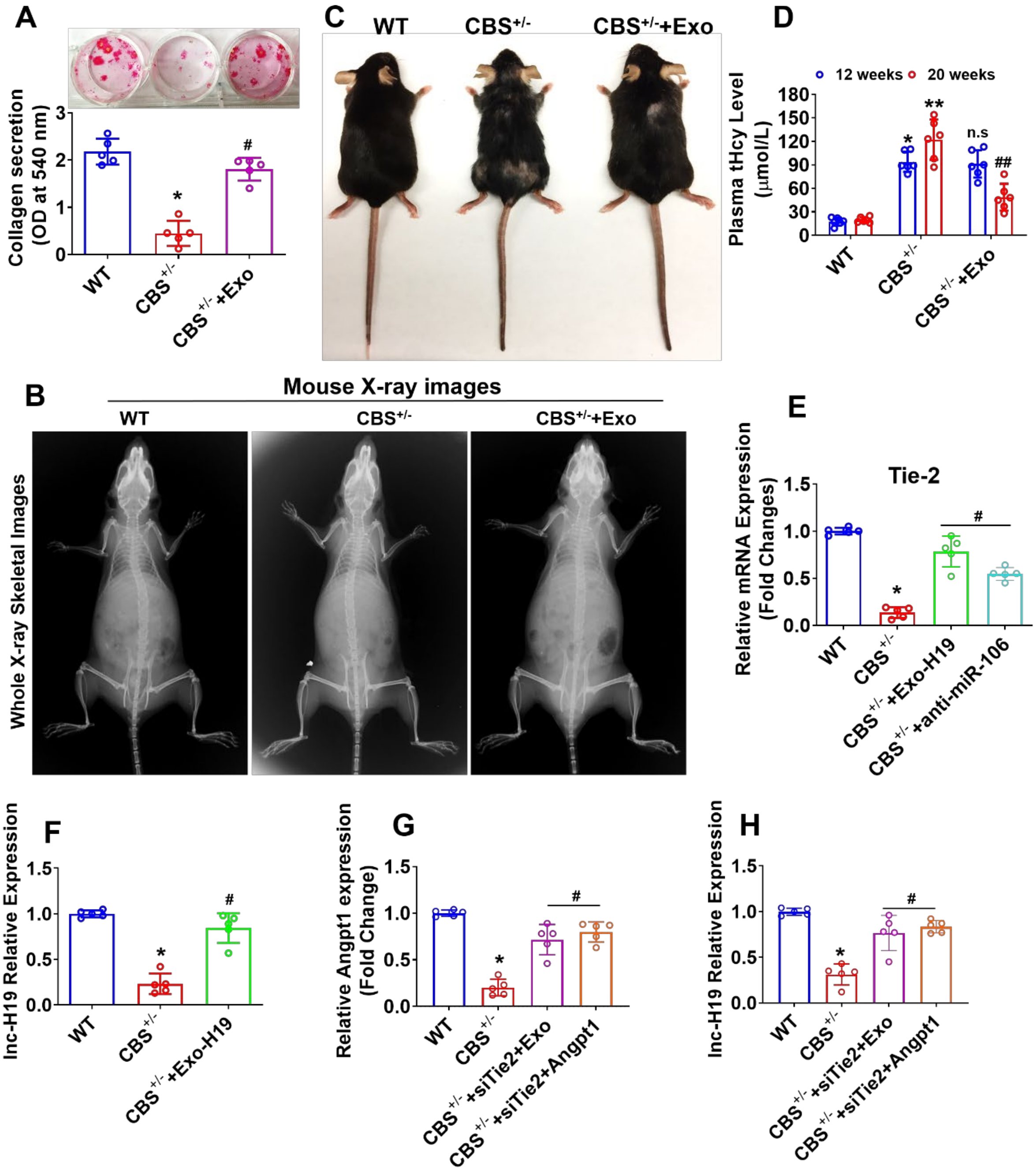


**Supplementary Figure S5. Exo effect on collagen formation and skeleton.** (A) Sirius Red dye staining of the collagen matrix of BMMSCs culture treated with Exo at days 14. (B) X-ray images of the whole skeleton of experimental mice. (C) Representative view of 14-weeks-old WT, $CBS^{+/-}$ and $CBS^{+/-}$+Exo mice. (D) Total homocysteine (tHcy) was measured in plasma of experimental mice following transplantation of Exo for 8-weeks. (E) Tie-2 mRNA transcript expression was

studied under Exo-H19 treatment in BMMSCs by qPCR analysis. (F) H19 expression was performed in BMMSCs using qPCR analysis. (G-H) Angpt1 and H19 expression was quantified in BMMSCs culture using qPCR assay. All data are expressed as mean±SEM. n=5-7 mice for all groups. *p< 0.05 compared with the WT-BMMSC and #p< 0.05 compared with the $CBS^{+/-}$-BMMSC. Supplementary Figure S5a: Exo were treated at the concentration of 100 μg to obtain the data from an *in vitro* culture experiment. Supplementary Figure S5b, c, d: Exo were treated at the concentration of 100 μg to obtain the data from an *in vivo* experiments.

**Supplementary Table S1: Sequences of PCR primers used for real-time quantitative PCR**

| **Gene Name** | **Primer sequence 5'-3'** |
|---|---|
| Runx2 | FP: TTTAGGGCGCATTCCTCATC<br>RP: TGTCCTTGTGGATTGAAAGGAC |
| Bglap | FP:GCGCTCTGTCTCTCTGACCT<br>RP: ACCTTATTGCCCTCCTGCTT |
| Angpt1 | FP: TGC AGC AAC CAG CGC CGA AA<br>RP: CAG GGC AGT TCC CGT CGT GT |
| Tie-2 | FP:GATTTTGGATTGTCCCGAGGTCAAG<br>RP:CACCAATATCTGGGCAAATGATGG |
| Ki67 | FP:AATCCAACTCAAGTAAACGGGG<br>RP:TTGGCTTGCTTCCATCCTCA |
| GAPDH | FP: TGCACCACCAACTGCTTGC<br>RP: GGCATGGACTGTAGTCAGAG |
| LncRNA-H19 | FP: ATCGGTGCCTCAGCGTTCGG<br>RP:CTGTCCTCGCCGTCACACCG |
| miRNA-106 | FP:GATGCTCAAAAAGTGCTTACAGTGCA<br>RP:TATGGTTGTTCTGCTCTCTGTCTC |
| miRNA-17-3p | FP:CCCGGGGAGCTCTAAAAATTGCACACA<br>RP:GGGCCCACGCGTCTATCTTGCGCTCCTG |
| miRNA-20b | FP:GAGCTCTAAAAATTGCACACACT<br>RP:CGTCTATCTTGCGCTCCTGAAAAACT |
| U6snRNA | FP:CTCGCTTCGGCAGCACA<br>RP:AACGCTTCACGAATTTGCGT |

**Footnotes:** Runt-related transcription factor 2 (RUNX2), bone gamma-carboxyglutamic acid-containing protein (Bglap), Angiopoietin 1 (Angpt1)

**Supplementary Table S2: Primary antibodies used**

| Antibody | Clone | Dilution | Source | Cat. No |
|---|---|---|---|---|
| **Western blot** | | | | |
| Rabbit Anti-RUNX2 antibody | polyclonal | 1:250 | Abcam | ab23981 |
| Rabbit Anti-Osteocalcin antibody | | 1:250 | Abcam | ab93876 |
| Mouse anti-Tie-2 Antibody | (3A5) | 1:200 | Santacruz Biotechnology | sc-293414 |
| Rabbit Phospho-Tie2 (Tyr992) Antibody | polyclonal | 1:250 | Cell signaling | #4221 |
| Mouse anti-eNOS Antibody | Monoclonal | 1:250 | Abcam | Ab76198 |
| Rabbit Phospho-eNOS (Ser1177) Antibody | polyclonal | 1:250 | Cell signaling | #9571 |
| Rabbit Anti-CD63 antibody | polyclonal | 1:200 | Abcam | Ab134045 |
| Rabbit Anti-CD9 antibody | Monoclonal | 1:250 | Abcam | Ab2215 |
| **Flow cytometry and immunofluorescence** | | | | |
| FITC Anti-mouse CD73 antibody | TY/11.8 | 1:200 | Biolegend | 127219 |
| FITC Rat IgG1, κ Isotype Ctrl Antibody | RTK2071 | 1:200 | Biolegend | 400405 |
| PE anti-mouse/human CD44 Antibody | IM7 | 1:200 | Biolegend | 103023 |
| PE Rat IgG2b, κ Isotype Ctrl Antibody | RTK4530 | 1:200 | Biolegend | 400607 |
